# ATF4 destabilizes RET through nonclassical GRP78 inhibition to enhance chemosensitivity to bortezomib in human osteosarcoma

**DOI:** 10.7150/thno.36818

**Published:** 2019-08-14

**Authors:** Jie Luo, Yuanzheng Xia, Yong Yin, Jun Luo, Mingming Liu, Hao Zhang, Chao Zhang, Yucheng Zhao, Lei Yang, Lingyi Kong

**Affiliations:** Jiangsu Key Laboratory of Bioactive Natural Product Research and State Key Laboratory of Natural Medicines, School of Traditional Chinese Pharmacy, China Pharmaceutical University, Nanjing 210009, China

**Keywords:** ATF4, RET, GRP78, bortezomib, osteosarcoma oncogenesis

## Abstract

**Rationale**: Activating transcription factor 4 (ATF4) is a central regulator of the cellular stress response and reduces tumor burden by controlling the expression of target genes implicated in the induction of apoptosis. Evidence shows ATF4 activation is responsible for proteasome inhibitor bortezomib (BTZ)-induced osteosarcoma (OS) cell death. However, it remains unclear how such suppressive function is impaired during prolonged therapeutic interventions.

**Methods**: Stable cells and* in vivo* xenograft models were generated to reveal the essential role of ATF4 in cell apoptosis and tumor growth. Fluorescence in situ hybridization (FISH) and immunohistochemistry were employed to detect the expression and significance of ATF4 in the specimens from osteosarcoma patients. Biochemical differences between chemoresistant and chemosensitive cancer cells were determined by proliferation, apoptosis, real-time PCR, immunoblotting and immunofluorescence. Promoter activity was analysed using the luciferase reporter assay. Immunoprecipitation was used to explore the interaction of proteins with other proteins or DNAs.

**Results**: ATF4 significantly inhibited OS tumorigenesis, whereas knockdown of ATF4 prevented the antitumor effects of BTZ. Normal osteoblasts are supposed to preferentially express ATF4, but *ATF4* silencing was detected in both OS clinical samples and BTZ-resistant sublines (OS/BTZ). We found that ATF4 downregulation was tightly linked to the aberrant expression of RET, primarily due to RET stabilization in OS/BTZ cells. Loss of RET upregulated ATF4 and potentiated the apoptotic response to BTZ. ATF4 recognized the TK domain of RET by recruiting its transactivated E3 ligase Cbl-c to accelerate RET proteasomal turnover, which in turn prevented BTZ resistance. In contrast, the chaperone GRP78 bound to RET and interfered with ATF4/RET interactions, promoted RET stabilization. Intriguingly, ATF4 repressed GRP78 transcription in OS/BTZ cells via the first ERSE, instead of transactivating GRP78 in wild-type OS via classical CRE element, revealing a dual targeting of RET and GRP78 to overcome chemoresistance.

**Conclusion**: The results uncover a crucial role for ATF4 in blocking the progression and resistance response in RET/GRP78-positive human osteosarcoma.

## Introduction

Osteosarcoma (OS) is the most common primary carcinoma of the bone that mostly occurs in teenagers and young adults 1. Clinical treatment modalities and outcomes for OS have not changed substantially over the past 30 years 2. Therefore, a better understanding of the molecular mechanisms underlying OS oncogenesis and the identification of novel therapeutic targets are urgently needed to improve the survival of OS patients.

As a stress-induced transcription factor, activating transcription factor 4 (ATF4) is frequently upregulated in a variety of cancers and is associated with resistance to proteasome inhibitors and chemotherapeutic agents 3, 4. However, accumulating evidence supports a model in which ATF4 is not merely a pro-tumorigenic effector but contextually switches to a pro-apoptotic signalling role for the formation of heterodimers with C/EBP homologous protein (CHOP) 5, 6. In contrast, our previous study and others have consistently reported that ATF4 can induce apoptosis in cancer cells irrespective of CHOP 7, 8. Moreover, stress-inducible chaperone glucose-regulated protein 78 (GRP78) has been implicated in resistance to chemotherapeutics 9. Abrogating BTZ-induced upregulation of GRP78 was showed to strengthen the effect of ATF4 in OS 7. The outcome of ATF4 activation is therefore highly context-dependent. Given the evidence for the low basal expression of ATF4 in OS cell lines under normal physiological conditions plus the upregulation of ATF4 in proteasome inhibitor bortezomib (BTZ)-induced OS cells 7, further studies are needed to uncover the distinct role of ATF4 in determining OS fate in response to BTZ therapy.

Recent advancements point to a role for ATF4 mutations in mediating drug resistance in tumor cells featured by xCT overexpression 10. Similarly, ATF4 has been proposed as a potential biomarker positively related to the efficacy of BTZ treatment 11. The results suggest that lower expression of ATF4 is correlated with shorter progression-free survival in multiple myeloma (MM) patients. Similarly, loss of function of ATF4 plays a role in initiation of medullary thyroid cancer (MTC) 12. This dependency prompted us to focus on whether ATF4 activation might also be a potent vulnerability for OS to perturbation after prolonged chemotherapy. RET is a receptor tyrosine kinase (RTK) that is required for normal cell development 13. Activation of RET occurs via oncogenic mutations in multiple sporadic carcinomas, most notably those of the thyroid and lungs 14, 15. Therapeutic approaches targeting RET with small-molecule kinase inhibitors are being evaluated for cancers that are associated with RET mutations or increased RET expression, such as MTC, chronic myelomonocytic leukaemia (CMML), and breast and pancreatic cancers 16-18. Importantly, Bagheri-Yarmand *et al.* 19 reported that RET contributes to cancer cell viability via the direct repression of ATF4 at the promoters of pro-apoptotic targets *NOXA* and *PUMA*. Despite the well-documented pathogenic, diagnostic and prognostic roles of RET in MTC 20, its precise role in BTZ-induced apoptotic cell death in OS remains unknown. ATF4 was also identified as a negative regulator of RET in MTC 12. However, the mechanistic link between ATF4 and RET signalling in OS development are still unclear.

Here, we determined the expression profiles of *ATF4* in two pairs of parental and BTZ-induced chemoresistant human OS cell lines (OS/BTZ) and clinical samples from OS patients. Genetic silencing of *ATF4* was observed in the OS/BTZ cells and cases. Based on these findings, we further elucidated the possible mechanisms of *ATF4* depletion underlying BTZ resistance. Our data revealed a conceptually novel functional link between ATF4 activation and RET stability in OS and clearly showed therapeutic benefits of ATF4 expression with rapid response to BTZ treatment.

## Materials and Methods

### Cell lines, cell culture, and reagents

The cell lines HEK293T, U-2 OS and HOS were purchased from the Cell Bank of the Chinese Academy of Sciences (Shanghai, China), and were characterized by Genetic Testing Biotechnology Corporation (Suzhou, China) using short tandem repeat (STR) markers. All cells were cultured in the recommended media supplemented with 10% foetal bovine serum, 100 U/mL penicillin and streptomycin at 37 °C in 5% CO_2_. BTZ-resistant cancer cells were obtained by a stepwise increase in the concentration of BTZ. U-2 OS and HOS cells were incubated with 5 nM BTZ for 2 days in the beginning. Then, the medium was changed to fresh medium without BTZ, and the cells were cultured until they grew well. At each subculture, the cells were incubated with gradually increasing concentrations of BTZ for 2 days. Some aliquots of cells were stored during the process. Cells that grew in the presence of the maximum concentration (U-2 OS, 200 nM; HOS, 150 nM) of BTZ were stored for further analyses. Stable ATF4-overexpressing or ATF4 knockdown cells, including ATF4-U-2 OS, ATF4-HOS, shATF4-U-2 OS, shATF4-HOS or their corresponding controls, were generated by the stable transfection of ATF4 EF1a-GFP/puro or shATF4 pGLV-h1-GFP/puro lentiviral vector (GenePharma, China), respectively. For the selection of stable clone cells, puromycin (2 μg/mL) was used. Recombinant human GDNF was purchased from R&D Systems Europe Ltd. BTZ, CBZ and MG132 were purchased from MedchemExpress (Monmouth Junction, NJ). CHX was purchased from APExBIO (Houston, USA).

### Xenografts

Five-week-old male athymic nude mice (nu/nu) were purchased from the Model Animal Research Center of Nanjing University (Nanjing, China) and maintained under pathogen-free conditions. Vector- or scrambled shRNA control-transfected and stable ATF4-overexpressing or ATF4-deficient HOS cells were harvested and resuspended in PBS. A total of 2 × 10^6^ cells in 100 µL PBS were subcutaneously injected into the rear flanks of mice. When tumors reached 100 mm^3^, the mice were divided into eight groups (six mice per group) and subjected to the following treatments: (1) control vehicle or (2) BTZ (1.0 mg/kg) every 3 days. Tumor volumes were measured with a caliper and calculated as 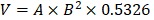
(A = long axis, B = short axis). At the end of the experiments, the mice were anaesthetized with isoflurane and sacrificed by cervical dislocation, and the tumors were harvested and weighed. All animals were maintained and used in accordance with the guidelines of the Institutional Animal Care and Use Committee (IACUC) of China Pharmaceutical University Experimental Animal Center.

### Fluorescence in situ hybridization (FISH) and immunohistochemistry

FISH was performed to detect ATF4 expression in human osteosarcoma and adjacent tissues (Servicebio, China), which was conducted as described previously 21. The ATF4 probe 5'-FAM-CATCCACAGCCAGCCATTCGG-FAM-3' was synthesized. A semi-quantitative scoring criterion was used for FISH, wherein both the staining intensity and positive cell number were recorded. Protein expression levels of xenografts from the mouse model were determined by standard immunohistochemistry protocols. Sections were scanned, and the images were then digitalized. Image-Pro Plus 5.1 software was used to calculate the integrated optical density (IOD) of the indicated mRNAs and proteins.

### Small interfering RNAs and antibodies

siRNA duplexes against human ATF4, RET, GRP78 and Cbl-c were obtained from R&S Biotechnology Co., Ltd. (Shanghai, China). Short hairpin RNAs against human ATF4 were purchased from GenePharma. Cells grown to a confluence of 50-70% were transfected with siRNAs using Lipofectamine 3000 (Invitrogen, CA) and plated again for subsequent experiments. Knockdown efficiency was determined using qRT-PCR or western blotting. As ATF4 protein levels were hardly detectable in OS parental and BTZ-resistant cell lines, shRNA-mediated knockdown efficiency was performed using their stable ATF4-transfected clones. Antibodies against FLAG, Myc and HA tags were purchased from Sigma-Aldrich (St. Louis, MO). Antibodies against ATF4, GRP78, RET, phospho-AKT (Ser473), AKT, phospho-ERK1/2 (Thr202/Tyr204), ERK1/2, ABCB1, MRP1, Bcl-2, cleaved-Caspase-9 (Asp315), cleaved-Caspase-3 (Asp175), cleaved PARP (Asp214), PARP, ubiquitin and GAPDH were purchased from Cell Signaling (Danvers, MA). Anti-*α*-tubulin were purchased from BOSTER (Wuhan, China). Antibodies against ATF4, RET and GRP78 for immunofluorescence and antibodies against BCRP, phospho-RET (Y1062) and Cbl-c were purchased from Abcam Inc. (Cambridge, MA). Anti-Bcl-2 and anti-PCNA antibodies from Bioss (Beijing, China) were used for immunohistochemical experiments. Anti-RET (C-3) and anti-GDNF (B-8) antibodies were purchased from Santa Cruz Biotechnology, Inc. (Santa Cruz, CA). Details regarding the dilutions of antibodies used are presented in **Table S1**.

### Immunoprecipitation and western blotting

Expression constructs (Myc-tagged full-length RET, RET-G93S, C634W, K758M, M918T, ΔTK, ΔCLD1, FLAG-tagged full-length ATF4, ATF4-ΔDBD, GST-ATF4, His-ATF4, HA-GRP78, and HA-Cbl-c) were cloned into the pMT/V5 vector according to standard cloning procedures. Transfection of plasmids and siRNAs was carried out with the Lipofectamine 3000 Transfection kit (Invitrogen, MA) following the manufacturer's protocol. IP and WB were performed using our published protocol 7. EasySee® Western Blot Kit from TransGen Biotech (Beijing, China) was used for protein detection.

### RNA extraction and quantitative RT-PCR

Total RNA was isolated using the EASYspin Plus tissue/cell RNA extraction kit (Aidlab, China) according to the manufacturer's protocol. One microgram of total RNA was reverse transcribed using the HiScript Q RT SuperMix kit (Vazyme, China). Quantitative PCR was then performed using TaqMan polymerase with SYBR Green fluorescence (Nippon Gene, Japan) on a LightCycler 480 Detector (Roche, Germany). Target gene threshold cycles (Ct values) were normalized to *GAPDH* as an endogenous control. The primer sequences are listed in **Table S2**.

### Cell proliferation and MTT assay

For the monolayer colony formation assay, cells were seeded in 12-well plates in triplicate (2.5 × 10^5^ cells/well). After overnight culture, the cells were transfected with equal amounts of the indicated expression plasmids or shRNA duplexes and the corresponding controls for 24 h using Lipofectamine 3000 (Invitrogen, CA), followed by BTZ (100 nM) or vehicle treatment for 24 h. The cells were selected for 12 days in medium containing 10% FBS and 500 μg/mL G418. For the clonogenic assay to detect BTZ resistance, cells were continuously cultured in medium containing 10% FBS and 100 nM BTZ for 2 weeks. The surviving colonies were stained with 0.1% crystal violet, and colonies with more than 50 cells were manually counted.

Cell viability was evaluated by MTT assays and evaluated using an ELISA reader (Spectra Max Plus384, Molecular Devices, Sunnyvale, CA). Relative cell viability was calculated based on the absorbance of untreated cells.

### Flow cytometry

Apoptosis was measured via flow cytometry. Briefly, after specific treatment, cells were pelleted and washed with PBS before being stained with Annexin V-fluorescein isothiocyanate (FITC) and propidium iodide. Apoptotic cells were analysed using a BD FACSCalibur flow cytometer (Becton & Dickinson Company, Franklin Lakes, NJ), and data were quantified using FlowJo 10.2 software. All experiments were performed three times, and statistical analysis (mean ± SD) from three separate experiments is shown.

### Immunofluorescence

Cells were fixed in 4% paraformaldehyde for 15 min and then incubated in 5% bovine serum albumin (BSA) with 0.1% Triton X-100 for 1 h to permeabilize the cells and block nonspecific protein-protein interactions. The cells were then incubated with the indicated antibodies (the dilution ratio selected was dependent on the product datasheets) overnight at 4°C. Alexa Fluor® 488- or 594-conjugated goat anti-rabbit IgG polyclonal antibody (1:200 dilution, Cell Signaling) was used as the secondary antibody. DAPI was used to stain cell nuclei. Immunofluorescence results were visualized using an ImageXpress Micro® system (Molecular Devices, CA).

### Molecular docking assay of ATF4 and RET

To study how ATF4 and RET interact with each other, we performed a protein-protein docking experiment using BioLuminate, Schrödinger's biologics modelling platform 22. The crystal structures of ATF4 (1CI6) 23 and RET (4CKJ, 5FM3) 24, 25 are available from Protein Data Bank 26. All protein structures were downloaded and prepared using the Protein Preparation Wizard in the Schrödinger Suite 2018-3 release. Default parameters were used for the docking step; ATF4 was selected as the ligand, and the two other molecules as receptors. The predicted change in binding affinity was calculated using the equation and thermodynamic cycle. We evaluated docking results using clustering analysis and weighted model scores as described by Decha et al. 27 From the top 30 cluster populations, we selected the binding pose that yielded the best average cluster weighed score (1CI6:4CKJ, second-rank conformation; 1CI6:5FM3, first-rank conformation). Docking images were visualized using Discovery Studio 2017 R2 Client.

### Luciferase assay

Plasmids encoding the luciferase reporter gene under the control of the GRP78 promoter were purchased from General Biosystems (Anhui, China) and were purified using the High Pure Plasmid Miniprep Kit (Real-Times, China). Parental or BTZ-resistant osteosarcoma cells were transfected using Lipofectamine 3000 (Invitrogen, CA) with either pGL3-basic or the mentioned wild-type and mutant GRP78 luciferase reporter plasmids together with ATF4 expression plasmids or shATF4 and the Renilla luciferase reporter pRL-TK vector (Promega, USA) as the reference control. Cells were subjected to luciferase activity measurement at 48 h post-transfection as described in the Dual-luciferase Reporter Assay kit (Promega, USA). All experiments were performed three times in triplicate. Representative data are shown.

### Chromatin immunoprecipitation and GST pull-down assays

ChIP assays were performed as previously described 28 with modifications. Formaldehyde-fixed cells were immunoprecipitated overnight with anti-ATF4 antibody or rabbit IgG. Quantitative real-time PCR with SYBR Green was used to determine the enrichment of immunoprecipitated material relative to input with gene-specific primers at the specified regions (**Table S2**). For GST pull-down assays, GST alone or recombinant GST-ATF4 immobilized on glutathione-agarose beads was incubated with U-2 OS cell extracts at 4 °C for 2 h. The beads were washed extensively, and the pulled-down proteins were mixed with SDS-PAGE sample buffer for analysis by immunoblotting with appropriate antibodies.

### Compound Library Screen

The established U-2 OS/BTZ cell line was screened against 1,452 compounds from an FDA-approved drug library (SelleckChem, Houston, USA) to identify potent ATF4 inducers. Cells were plated in 96-well plates and treated with vehicle (0.01% DMSO) or the compound library (average compound concentration of the library in medium was 10 µM). After a one-day incubation with the drug library, ATF4 protein levels were detected by immunofluorescence.

### Statistical analysis

Statistical analysis was performed with ANOVA or Student's t-test using GraphPad Prism version 5.0 (San Diego, CA). The data are presented as the mean ± SD or mean ± SEM. Generally, all experiments were carried out with n ≥ 3 biological replicates. Statistically significant differences of *P* < 0.05, *P* < 0.01 and *P* < 0.001 are noted with * or #, ** and ***, respectively.

## Results

### ATF4 overexpression decreases OS tumor burden

**Figure 1:**
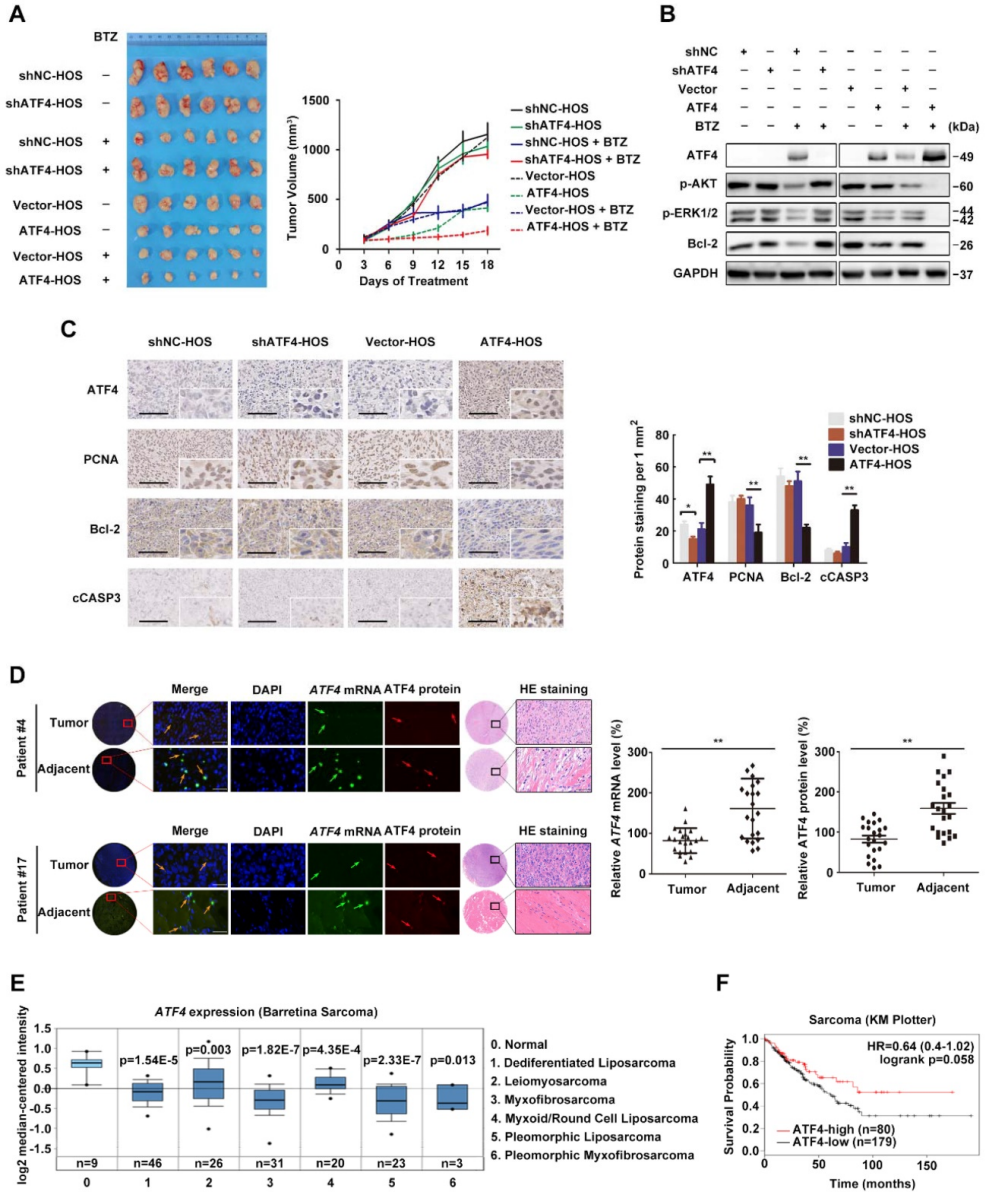
**High ATF4 expression is correlated with positive outcomes of osteosarcoma patients**.** A,** Growth of tumors derived from HOS cells with ATF4 alterations implanted subcutaneously in athymic mice in the absence or presence of BTZ (1.0 mg/kg). Tumor size was recorded every 3 days. Data are represented as the mean ± SEM from six mice. **B,** Homogenates of three representative tumors from each group after 18 days of treatment were analysed by western blotting to detect the levels of ATF4, p-AKT, p-ERK1/2 and Bcl-2. **C,** Representative images of immunohistochemical staining for ATF4, PCNA, Bcl-2 and cleaved-Caspase-3 (cCASP3) in the indicated mouse xenografts are shown. Microvessel density (MVD) of positively stained vessels were counted at 400×. Scale bars, 10 μm. Quantification of the indicated protein expression in mice tumors using Image-Pro Plus 6.0 software. **D,** Detection of abnormalities in *ATF4* mRNA (green) and protein (red) in 22 human osteosarcoma tumor and adjacent nontumor tissues (with HE staining; scale bars, 100 μm) using fluorescence in situ hybridization (FISH) plus immunohistochemistry. Scale bars, 50 μm. **E,** Box plots showing differential *ATF4* mRNA expression levels between sarcoma and normal soft tissues in Oncomine datasets. Error bars, SD. **F,** Kaplan-Meier curves showing the overall survival of patients with sarcoma (KM plotter database) in terms of *ATF4* gene expression.

We previously identified ATF4 as being required for the sensitivity of OS to ER stress-induced apoptosis in response to the proteasome inhibitor BTZ 7. To assess the *in vivo* effect of ATF4 alterations in xenografts, we implanted HOS cells stably expressing ATF4 and conditional shRNAs against ATF4 into the flank of BALB/c mice and intraperitoneally treated the mice with either BTZ or vehicle. ATF4 overexpression markedly decreased the tumor burden, which was comparable to the effect of BTZ. After 18 days of treatment, the growth of ATF4-expressing xenografts in BTZ-treated animals remained nearly completely inhibited, whereas the growth of xenografts of vehicle-treated animals was significantly more pronounced (median tumor volume increase 5.3% ± 27.8% versus 549.3% ± 133.5%; *P* = 0.001; **Figure 1A**). In contrast, the growth of ATF4-deficient xenografts was not susceptible to BTZ *in vivo* (**Figure 1A**). At necropsy, we observed no difference between BTZ- and vehicle-treated animals in the size of ATF4-deficient heterotopic tumors or expression of p-AKT, p-ERK1/2, or Bcl-2 within the tumors (**Figure 1B**). Concomitantly, we observed that the suppression of RAS-MAPK and PI3K-AKT signalling pathways after BTZ therapy was partially reversed *in vivo* by ATF4 depletion, as indicated by the restoration of p-AKT and p-ERK1/2. Furthermore, the phosphorylation of AKT and ERK was almost exactly eliminated in endogenous ATF4-expressing xenografts treated with BTZ **(Figure 1B)**. There were fewer Bcl-2- or PCNA-positive cells in the ATF4-expressing tumors than in the control group (**Figure 1C**).

We further examined whether ATF4 could induce tumor cell apoptosis. HOS xenografts stably expressing ATF4 were tested using the TUNEL assay. The results showed that compared with the control, ATF4 expression significantly increased the number of apoptotic HOS cells *in vivo* (from 2.2 ± 1.3% to 18.7% ± 3.2%; *P* = 0.001; **Figure S1A**). Next, we screened several OS cell lines in which endogenous ATF4 was silenced using shRNA or overexpressed using plasmid constructs and examined clonogenic survival (**Figure S1B**). Consistent with *in vivo* models, OS cells showed increased survival in response to BTZ when endogenous ATF4 was silenced; however, ATF4 overexpression alone not only suppressed the proliferation of tumor cells but also sensitized the cells to BTZ-induced growth arrest. The expression and subcellular localization of ATF4 in OS tissues from patients were investigated by fluorescence in situ hybridization (FISH) plus immunohistochemistry (**Figure 1D**). We found lower *ATF4* mRNA and protein expressions in OS tissues than those in adjacent normal tissues. Notably, RNA FISH analysis revealed an exclusively cytoplasmic and focal distribution of ATF4 in OS tissues, but simultaneous cytoplasmic and nuclear distribution of ATF4 in 86% (19/22) of the normal tissues tested, suggesting that ATF4 in OS tissues exhibits negligible functions both due to its deficiency and inactive status in the cytoplasm 29. Further bioinformatics analysis of public databases confirmed *ATF4* downregulation in other clinical sarcoma samples (**Figure 1E**). We also performed Kaplan-Meier survival analysis using an online database (http://kmplot.com). Low *ATF4* expression was associated with poor outcomes in patients with sarcoma (**Figure 1F**), suggesting an important role for ATF4 disruption in tumorigenesis.

### RET is elevated during the progression of BTZ resistance

**Figure 2:**
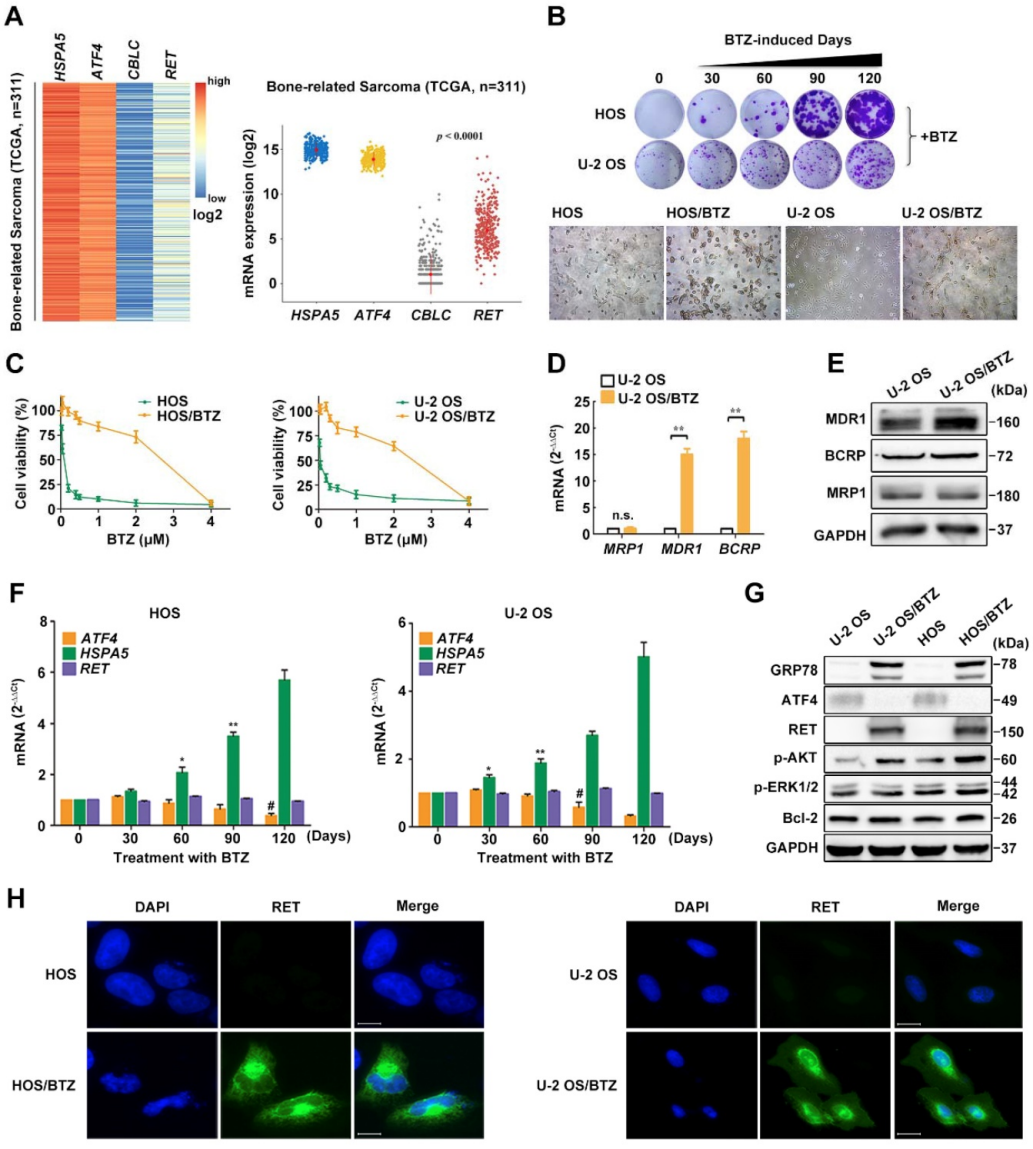
**Phenotypes of BTZ-resistant OS cell lines**. **A,** Heatmap of differentially expressed genes *HSPA5*, *ATF4*, *CBLC* and *RET* and their relative mRNA expression levels in 311 bone-related sarcoma samples from TCGA database. **B,** BTZ-resistant models of U-2 OS and HOS cells established at the indicated time were cultured in medium containing BTZ (100 nM) for two weeks. Then, the cells were fixed and stained (upper panel). Dramatic morphological changes were observed in BTZ-resistant tumor cells where the typical cobblestone-like appearance of wild-type OS cells was replaced by a spindle-like fibroblastic morphology (lower panel). **C,** BTZ-sensitive parental or resistant cell lines were treated with the indicated concentration of BTZ for 2 days. The growth suppressive effects of BTZ were measured by MTT analysis. **D and E,** The mRNA expression (**D**) and protein expression (**E**) of ABC transporters MRP1, MDR1 and BCRP in U-2 OS and U-2 OS/BTZ cell lines. **F,** Alterations in the *ATF4*, *HSPA5* and *RET* genes upon prolonged treatment with BTZ (U-2 OS, up to 200 nM; HOS, up to 150 nM). **G,** Differential analysis of the expression of indicated proteins in parental cells and BTZ-resistant sublines by immunoblotting. Data are representative immunoblots from three independent assays. **H,** Immunofluorescence analysis of RET protein (green) in OS/BTZ cells and parental cells. Nuclei were stained with Hoechst 33342 (blue). Images were captured using a high-content imaging system. Scale bars, 10 μm.

To explore the possible regulators implicated in ATF4 deprivation, we analysed the interrelated genes using KEGG PATHWAY database and STRING database 30 (https://string-db.org). A set of 19 genes was enriched for the reference group and represented a combination of pathways in cancer (hsa05200), protein processing in the ER (hsa04141) and ubiquitin-mediated proteolysis (hsa04120) (**Figure S2A**). We next examined the expression of 19 genes in different bone-related sarcomas using The Cancer Genome Atlas (TCGA) database (**Figure S3**) and found relatively high *HSPA5* and *ATF4* mRNA levels in tumor tissues and significantly low* CBLC* and *RET* expression levels (**Figure 2A and S2B**). Previous studies have well characterized the interaction between ATF4 and HSPA5 4. However, little is known about the role of ATF4 or HSPA5 in the RET signalling pathway in cancer (**Figure S2C**).

Our observation from RNA FISH in human OS and normal samples led us to hypothesize that ATF4 might undergo a specific inhibition upon prolonged chemotherapy 11, 31. To test this, we successfully developed *in vitro* BTZ-resistant models of HOS and U-2 OS sublines (**Figure S2D**). BTZ sensitivity remarkably decreased after prolonged treatment with BTZ (up to 150 nM, 4 months) (**Figure 2B**, upper panel; **Figure S2E**). Dramatic morphological changes were observed in the BTZ-resistant tumor cells (**Figure 2B**, lower panel). BTZ potently inhibited the viability of parental HOS and U-2 OS cell lines, with IC_50_ values of ~ 0.1 μM. In contrast, drug-resistant OS cells were resistant at doses up to ~ 2.5 μM (**Figure 2C**), indicative of a wide resistance index. We then identified abnormally expressed genes and proteins of the ATP-binding cassette (ABC) family in OS/BTZ cell lines 32. Remarkable increases in two important ABC transporters, MDR1 and BCRP, were observed in the BTZ-resistant U-2 OS cells at both mRNA and protein levels compared with that in their parental cells, consistent with prior reports of MDR1 overexpression as the most notable change in BTZ-resistant cancer cells 33-35 (**Figure 2D-E**). Additionally, ATF4 loss was detected in stable BTZ-resistant OS cells (**Figure 2F-G**).

To understand how the loss-of-function of ATF4 contributes to osteosarcoma resistance, we focused on the ATF4-responding proteins screened (**Figure S2B**). Given the strong link between GRP78 overexpression and the development of resistance to chemotherapeutic agents 36, 37 and the knowledge of BTZ as an ER stress stimulus 35, it was not surprising that GRP78 was progressively upregulated by BTZ induction (**Figure 2F-G**). Overexpression of specific RTKs was reported to correlate with metastasis and overall poor prognosis in human OS 38. We speculated that RTKs activation may also be involved in the molecular mechanisms of chemoresistance in OS. Here, we identified an unexpected activity of RET in OS/BTZ sublines, albeit undetectable in the context of cisplatin chemoresistance 39. Phosphorylation of AKT and ERK, components of RET downstream signal transduction pathways, was initiated (**Figure 2G**). Confocal microscopy analysis revealed ectopic expression of RET and GRP78 as characteristic features of BTZ-resistant OS cell lines (**Figure 2H and S2F**).

**Figure 3:**
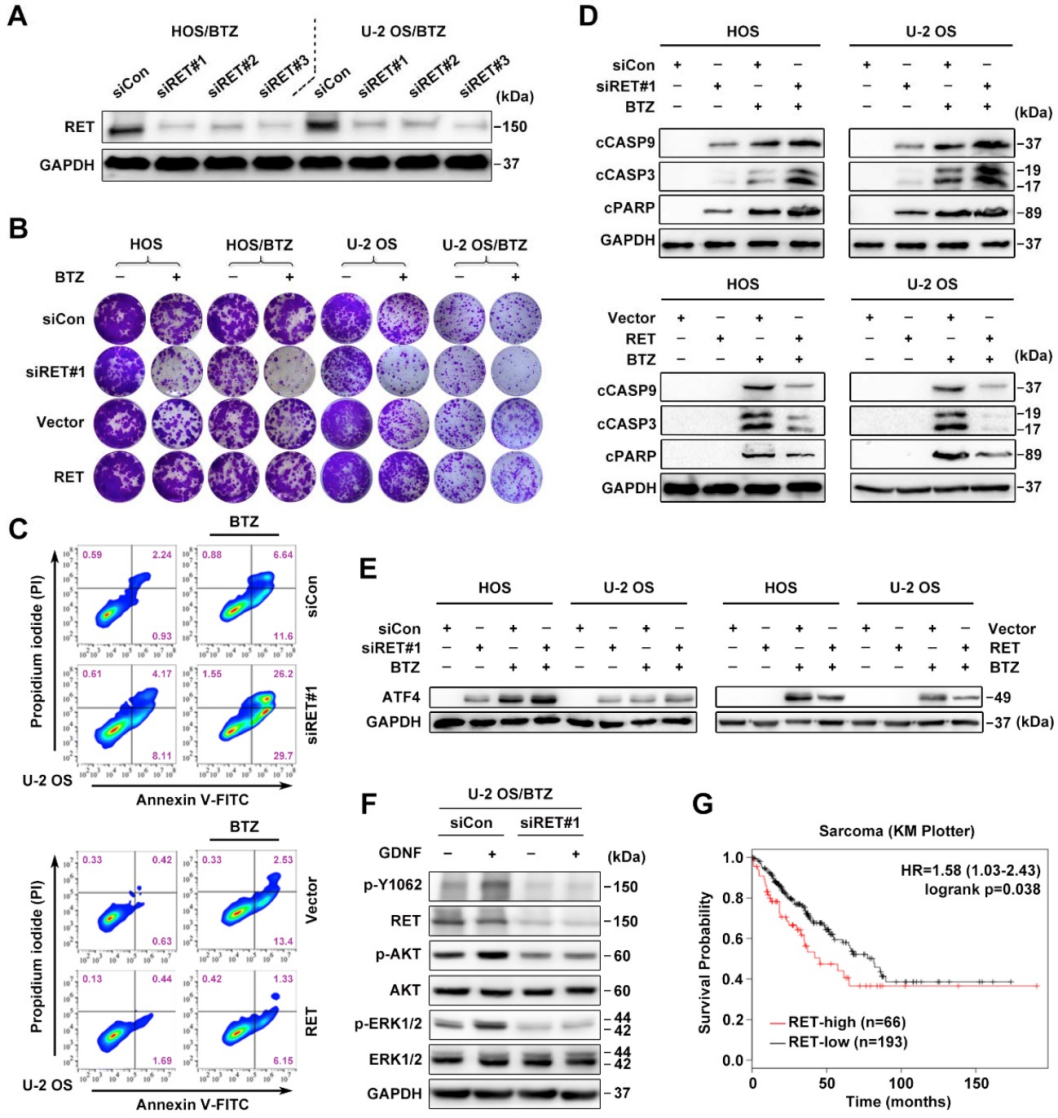
** RET is an important driver of OS tumorigenesis and confers resistance against BTZ**.** A,** Three individual siRNAs targeting RET were introduced into U-2 OS/BTZ and HOS/BTZ sublines by Lipofectamine transfection for 24 h. Lysates of control and RET knockdown BTZ-resistant cells were blotted to confirm RET suppression efficiency. **B,** Colony formation-promoting activity of RET. Control and RET knockdown (siRET#1) or RET-overexpressing OS or OS/BTZ cells were cultured for 2 weeks with BTZ (100 nM) treatment for the first two days of the experiment. Then, the cells were fixed and stained. **C,** RET protects cells against BTZ-induced apoptosis. U-2 OS_siRET#1_ and U-2 OS_siCon_ cells or U-2 OS_RET_ and U-2 OS_vector_ cells were challenged by either vehicle or BTZ (100 nM), stained with fluorescein isothiocyanate (FITC)-conjugated Annexin V and propidium iodide (PI), and analysed by flow cytometry to evaluate the role of RET in the prevention of apoptosis. **D,** RET blocks BTZ-induced activation of the caspase cascade. OS_siRET#1_ and OS_siCon_ cells or OS_RET_ and OS_vector_ cells were incubated with BTZ (100 nM) for 24 h to determine the levels of molecules involved in caspase pathways. **E,** Interference of RET with the antitumor effects of BTZ is connected with ATF4 inhibition as shown via western blot analysis of ATF4 expression. **F,** RET activation in BTZ-resistant OS cell lines. U-2 OS/BTZ OS_siCon_ and U-2 OS/BTZ_siRET#1_ cells cultured in serum-free medium overnight were stimulated for 30 min with or without 20 ng/mL GDNF and subjected to western blotting using the indicated antibodies. **G,** Kaplan-Meier curves showing survival benefits of high *RET* expression in patients with sarcoma (KM plotter database).

Expression and activation of wild-type RET is recognized in several tumor types 13. We therefore studied whether RET overexpression identified in OS/BTZ gives rise to a vulnerability that can be therapeutically exploited. To validate* RET* as a gene whose suppression confers sensitivity to BTZ, we introduced three small interfering RNAs (siRNAs) against *RET* (#1, #2 and #3) into OS cells by Lipofectamine-based transfection. Scrambled siRNA (siCon) served as a control. All three distinct *RET* siRNAs remarkably suppressed *RET* mRNA and protein expression (**Figure 3A and S2G**), sensitized both OS and OS/BTZ cells to BTZ (**Figure 3B and S2H**), and enhanced the growth inhibitory effect of the RTK inhibitor Cabozantinib (**Figure S2I**). Exogenously expressed RET, in turn, restored the resistance of these cells to BTZ (**Figure 3B and Figure S2J**). We next evaluated how RET modulates BTZ-induced apoptosis. U-2 OS cells undergoing RET alterations were challenged with either BTZ or vehicle, stained with fluorescein isothiocyanate (FITC)-conjugated Annexin V and propidium iodide (PI), and analysed using flow cytometry. Upon BTZ challenge, the percentage of Annexin V+/PI- siRET-transfected U-2 OS cells (U-2 OS_siRET_) showed a marked increase compared with U-2 OS_siCon_ cells (**Figure 3C**, upper panel; **Figure S4A**); there was also a significant difference in the percentage of Annexin V+/PI- cells between U-2 OS_RET_ and U-2 OS_Vector_ (**Figure 3C**, lower panel; **Figure S4A**). These data suggested that RET protects U-2 OS cells against BTZ-induced apoptosis. To assess the role of RET in the BTZ-induced activation of caspases, we challenged two groups of OS_siRET_ and OS_siCon_ cells with either BTZ or vehicle and subjected the cell lysates to immunoblot analysis (**Figure 3D**, upper panel). BTZ induced more cleavage of caspase-9, caspase-3 and PARP in OS_siRET_ cells than in OS_siCon_ cells. An inverse response was observed when we challenged OS_Vector_ and OS_RET_ cells with BTZ (**Figure 3D**, lower panel). In this system, RET prevented the activation of intrinsic caspase-dependent pathway in BTZ-challenged OS cells, contrary to the role of ATF4 in such apoptotic induction; and indeed, the activation of ATF4 was antagonized by RET overexpression with BTZ exposure (**Figure 3E**). Given that the glial cell line-derived neurotrophic factor (GDNF) is the major ligand of RET 18, we also examined the effect of GDNF on RET signalling hubs. An increase in phosphorylation at tyrosine 1062 (p-Y1062) of RET and the activation of AKT and ERK signalling pathways was detected after a transient treatment of U-2 OS/BTZ cells with GDNF, whereas pre-treatment with RET siRNAs completely blocked this response (**Figure 3F**), suggesting that GDNF might promote RET-mediated BTZ resistance. Online Kaplan-Meier survival analysis further demonstrated the oncogenic role of RET in sarcoma (**Figure 3G**).

### ATF4 promotes OS chemosensitivity via the inhibition of RET

**Figure 4:**
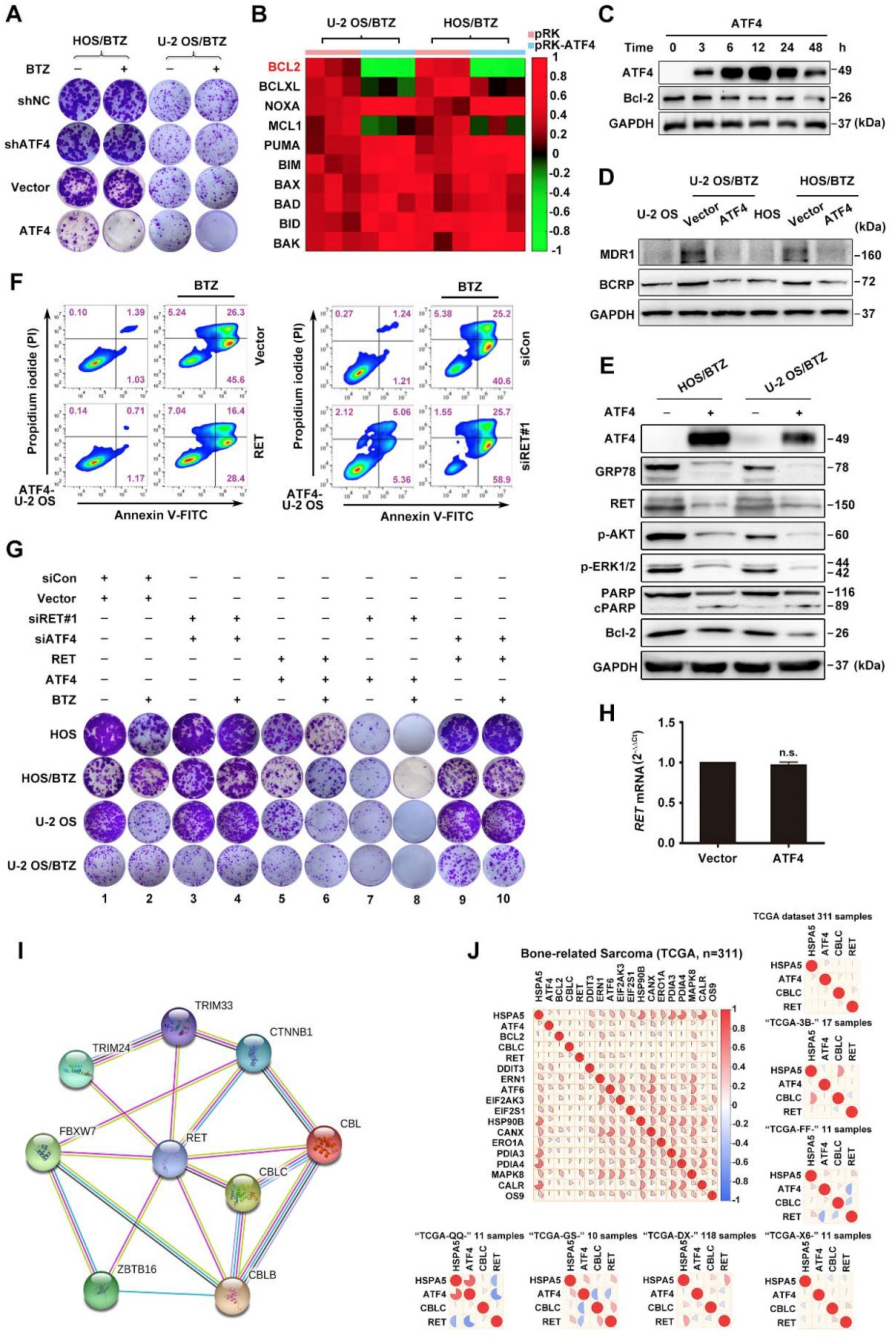
** The significance of crosstalk between ATF4 and RET in OS and OS/BTZ cells. A,** The functional phenotypes of retroviral shATF4 and ATF4-transfected OS/BTZ sublines are indicated by colony formation assays in 100 nM BTZ. The pGLV or EF1a vector was used as a control. The cells were fixed, stained, and photographed after 14 days. **B,** qRT-PCR analysis of the *BCL-2* family in OS/BTZ sublines challenged by the ectopic expression of ATF4. Relative Ct values 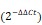 are indicated in different colours in the heatmap. **C,** Expression of ATF4 and Bcl-2 proteins in U-2 OS/BTZ_ATF4_ cells for the indicated time. **D,** Levels of MDR1 and BCRP were measured by immunoblotting in OS/BTZ_vector_ and OS/BTZ_ATF4_ cells and compared with those in parental cells. **E,** Comparative analysis of the expression of the indicated proteins in OS/BTZ_vector_ and OS/BTZ_ATF4_ cells. **F,** Stable U-2 OS_ATF4_ cells transfected with RET or siRET#1 were challenged with either vehicle or BTZ (100 nM), stained with FITC-conjugated Annexin V and PI, and analysed by flow cytometry to evaluate the role of RET in the prevention of apoptosis induction involving ATF4 overexpression. **G,** Colony formation assays in OS and OS/BTZ cells, with either ATF4 or RET alterations mediated by the transient transfection of siRNAs or expression vectors, cultured in 100 nM for the first two days of the experiment. The cells were fixed, stained, and photographed after 14 days.** H,** mRNA expression analysis of the *RET* gene in U-2 OS/BTZ_ATF4_ cells using qRT-PCR. Data were expressed as the mean ± SD (n = 3) and analysed by two-tailed unpaired t-tests. n.s., non-significant. **I,** *RET* and its associated genes implicated in ubiquitin pathways in the STRING database. **J,** Heatmap showing the correlation of the indicated genes in 311 bone-related sarcoma samples and subsets (n ≥ 10) in TCGA database.

Because ATF4 deficiency was observed in chemoresistant OS cells, we speculated that ATF4 expression might have a profound effect on the development of tumor resistance. Long-term cell proliferation assays showed that ATF4 overexpression markedly reversed BTZ resistance in OS/BTZ cells. Depletion of ATF4 rescued OS/BTZ sublines from the antiproliferative effects of BTZ (**Figure 4A and S4B**). BCL-2 family proteins are key regulators of programmed cell death 40. Based on these findings, we next investigated ATF4-mediated signalling of survival-promoting or apoptosis-promoting activities involving BCL-2 family genes in BTZ-resistant OS cells 41. A heatmap of all these genes involved in cell fate determination revealed various changes in their mRNA levels when ATF4 was overexpressed, with the most distinct downregulation of *BCL2* (**Figure 4B**), one prominent gene that is highly expressed in bone-related sarcoma samples (**Figure S2B**). Consistent with this, the protein level of Bcl-2 was markedly repressed over time in U-2 OS/BTZ cells upon transient expression of ATF4 (**Figure 4C**), suggesting its potential association with the establishment of chemosensitivity mediated by ATF4 activation.

Considering the overexpression of MDR1 and BCRP in OS/BTZ cells, we confirmed that aberrant ATF4 expression in BTZ-resistant OS cells effectively decreased the protein levels of MDR1 and BCRP to those in the wild-type cells (**Figure 4D**). Compared with untreated OS/BTZ cells, OS/BTZ_ATF4_ cells expressed low levels of the RET protein (**Figure 4E**) upon the simultaneous inactivation of downstream AKT and ERK pathways, suppression of GRP78 and Bcl-2 proteins, and upregulation of activated PARP. We next addressed the role of RET in stable ATF4-expressing U-2 OS cells concerning BTZ-induced apoptosis. Flow cytometry analysis revealed that compared with the control, RET overexpression significantly reduced the number of BTZ-mediated apoptotic ATF4-U-2 OS cells (**Figure 4F**, left; **Figure S4C**). Strong synergy was observed when RNAi-*RET* was combined with BTZ (**Figure 4F**, right; **Figure S4C**).

As inhibition of RET by RNAi resensitized OS_ATF4_ and OS/BTZ_ATF4_ cells to BTZ, we reasoned that RNAi-mediated RET knockdown should act synergistically with BTZ to inhibit proliferation in these cell lines. To test this hypothesis, we cultured parental and OS_ATF4_ or OS/BTZ_ATF4_ cells in the absence and presence of RNAi-*RET*/RET expression vector, BTZ, or a combination of both. Exogenously expressed RET in OS_ATF4_ and OS/BTZ_ATF4_ cells partially restored resistance to BTZ (**Figure 4G**, lane 1 versus lane 5, lane 2 versus lane 6, and lane 5 versus lane 6, respectively; **Figure S4D** combined with **Figure S1B**, **S2J and S4B**). Surprisingly, RNAi-*RET* alone largely prevented the growth of OS_ATF4_ and OS/BTZ_ATF4_ cells, and RNAi-*RET* plus BTZ almost completely inhibited cell proliferation (**Figure 4G**, lane 1 versus lane 7, lane 2 versus lane 8, and lane 7 versus lane 8, respectively; **Figure S4D** combined with **Figure S1B and S2H**). In contrast, such growth arrest appeared to not occur in OS_shATF4_ or OS/BTZ_shATF4_ cells regardless of the alterations in RET expression (**Figure 4G**, lane 3 versus lane 4, and lane 9 versus lane 10, respectively; **Figure S4D**). Thus, ATF4 activation is sufficient to reverse BTZ resistance in OS cells independent of RET.

### ATF4 interacts with RET and promotes its degradation

**Figure 5:**
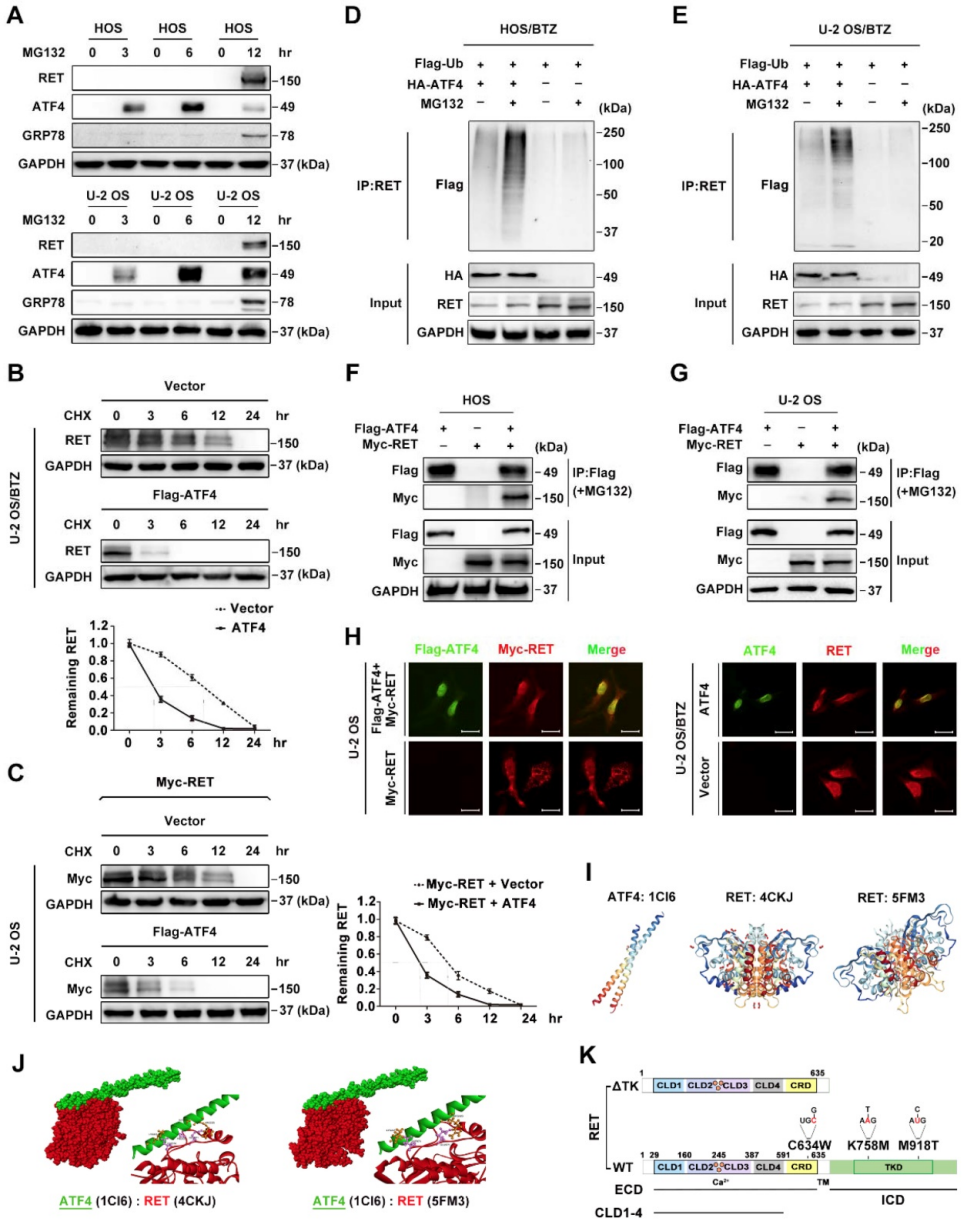
** Binding of ATF4 increases RET degradation**. **A,** Negative correlation of ATF4 and RET protein activation. Western blotting analysis of lysates from HOS and U-2 OS cells treated with the proteasome inhibitor MG132 (10 μM) for the indicated times. **B and C,** ATF4 reduces the half-life of the RET protein. U-2/BTZ cells and U-2 OS cells transfected with the FLAG-ATF4 vector (**B**) or cotransfected with the FLAG-ATF4 vector and Myc-RET vector (**C**) were treated with cycloheximide (CHX) (10 μM), and the expression of the indicated proteins was determined by immunoblotting at the indicated times. **D and E,** ATF4 mediates RET ubiquitination. HOS/BTZ (**D**) or U-2 OS/BTZ (**E**) sublines were cotransfected with plasmids encoding FLAG-ubiquitin (2 μg) and HA-ATF4 (4 μg). After transfection, the cells were incubated with or without MG132 (20 μM) for 4 h. Cell lysates were immunoblotted with the indicated antibodies.** F and G,** FLAG-ATF4 and Myc-RET were transfected into HOS (**F**) or U-2 OS (**G**) cells as indicated. Immunoprecipitated products were analysed by western blotting using the indicated antibodies to determine the presence of the appropriate proteins. The results represent the average from three independent experiments. Data are presented as the mean ± SD. **H,** ATF4 colocalizes with RET in the nucleus. After U-2 OS cells were transiently transfected with ATF4 (4 μg) and Myc-RET (4 μg) expression plasmids (left panel), or U-2 OS/BTZ sublines transiently were transfected with ATF4 (4 μg) vector (right panel), the cells were incubated in the presence of MG132 (20 μM) for 4 h and subsequently subjected to immunocytochemistry. The cells were stained with anti-ATF4 (green) and anti-RET (red) antibodies. Scale bars, 10 μm. **I,** Three-dimensional structures of ATF4 and RET^TK^ from PDB are shown.** J,** Intermolecular interactions between ASP974 and GLU971 at the TK domain of RET (red) with LYS335 and ARG323 of ATF4 (green). **K,** Schematic representation of full-length WT RET and various point mutants (C634W, K758M and M918T) or the truncation RET construct lacking the tyrosine kinase domain (TKD).

Significant differences in expression levels enabled us to investigate the crosstalk among *ATF4*, *HSPA5*, *CBLC* and *RET*. The results showed a correlation of the indicated genes in 311 sarcoma samples and the subsets (**Figure 4J**). We therefore set out to verify the data using a BTZ/OS model. Although RET is frequently undetectable in some human cancers, including OS 39, we found an overt accumulation of the RET protein without a change its mRNA level in response to prolonged treatment with the proteasome inhibitor BTZ (**Figure 2F-G**). Moreover, we observed that ATF4 did not affect *RET* mRNA level, suggesting the presence of posttranscriptional regulation (**Figure 4H**). Prior reports have revealed that the rapid degradation of RET is initiated by ubiquitination and predominantly carried out by proteasomes 42. Thus, we first investigated whether endogenous RET proteins are regulated by proteasomal degradation. Transient treatment of OS cells with the proteasome inhibitor MG132 did not result in detectable RET protein until the cells had been treated for 12 h. In contrast, ATF4 expression was promptly augmented in the presence of MG132 but attenuated markedly over time (**Figure 5A**). This suggested that RET is unstable and that ATF4 expression is highly correlated with RET protein accumulation. In addition, the half-life of RET in the BTZ-resistant U-2 OS cells transfected with FLAG-ATF4 plasmids was approximately 3 hours, whereas the half-life of RET in cells transfected with control vectors was as long as 9 hours (**Figure 5B**). Similar results were obtained when protein synthesis was inhibited using cycloheximide (CHX) after the transfection of U-2 OS cells with Myc-RET plus FLAG-ATF4 plasmids (**Figure 5C**). We next investigated the involvement of ATF4 in the ubiquitination and degradation of RET 43. As expected, ATF4 overexpression in stable BTZ-resistant OS cells significantly facilitated the ubiquitination of endogenous RET in a concentration-dependent manner (**Figure 5D-E and S5A**). To explore the mechanism by which ATF4 modulates RET stability, we examined the ability of ATF4 to interact with RET. In a coimmunoprecipitation assay with ectopically expressed ATF4 and RET, we found that Myc-tagged RET could precipitate FLAG-ATF4 from transfected OS cells (**Figure 5F-G**). This observation was further supported by immunofluorescence staining in both parental and chemoresistant U-2 OS sublines, showing that ATF4 colocalized with RET predominantly in the nucleus (**Figure 5H**). Together, these results suggested that ATF4 likely inhibits RET by mediating its degradation.

To further verify the binding of ATF4 to RET, we performed molecular docking experiments using techniques described in detail in the Methods section. Because of the multifunctional tyrosine kinase (TK) domain of RET, with its activation characterized by dimerization and autophosphorylation 18, a molecular docking study the interaction of both hyper-phosphorylated RET^TK^ (PDB ID: 5FM3) and wild-type RET^TK^ (PDB ID: 4CKJ) with ATF4 (PDB ID: 1CI6) was performed (**Figure 5I**). Two highest-scoring binding favourability models of the ATF4-RET interaction were discovered (**Figure 5J**). We then inspected the docking of the two complexes. We observed that the amino acid residues (ASP974 and GLU971) of two RET structures were positioned in close proximity to the binding surface of ATF4 (LYS335 and ARG323, respectively), suggesting a relatively stable docking conformation regardless of the RET phosphorylation status.

Given the computational docking results and the significance of clinical subtypes harbouring different *RET* oncogenic mutations 18, we continued to assess whether the TK domain of RET was necessary for ATF4 controlling its stability *in vitro*. First, we generated several deletion or point mutants of the Myc-tagged RET long isoform (RET 51) for coimmunoprecipitation with ubiquitin upon ectopic ATF4 expression in OS cells (**Figure 5K**).

**Figure 6:**
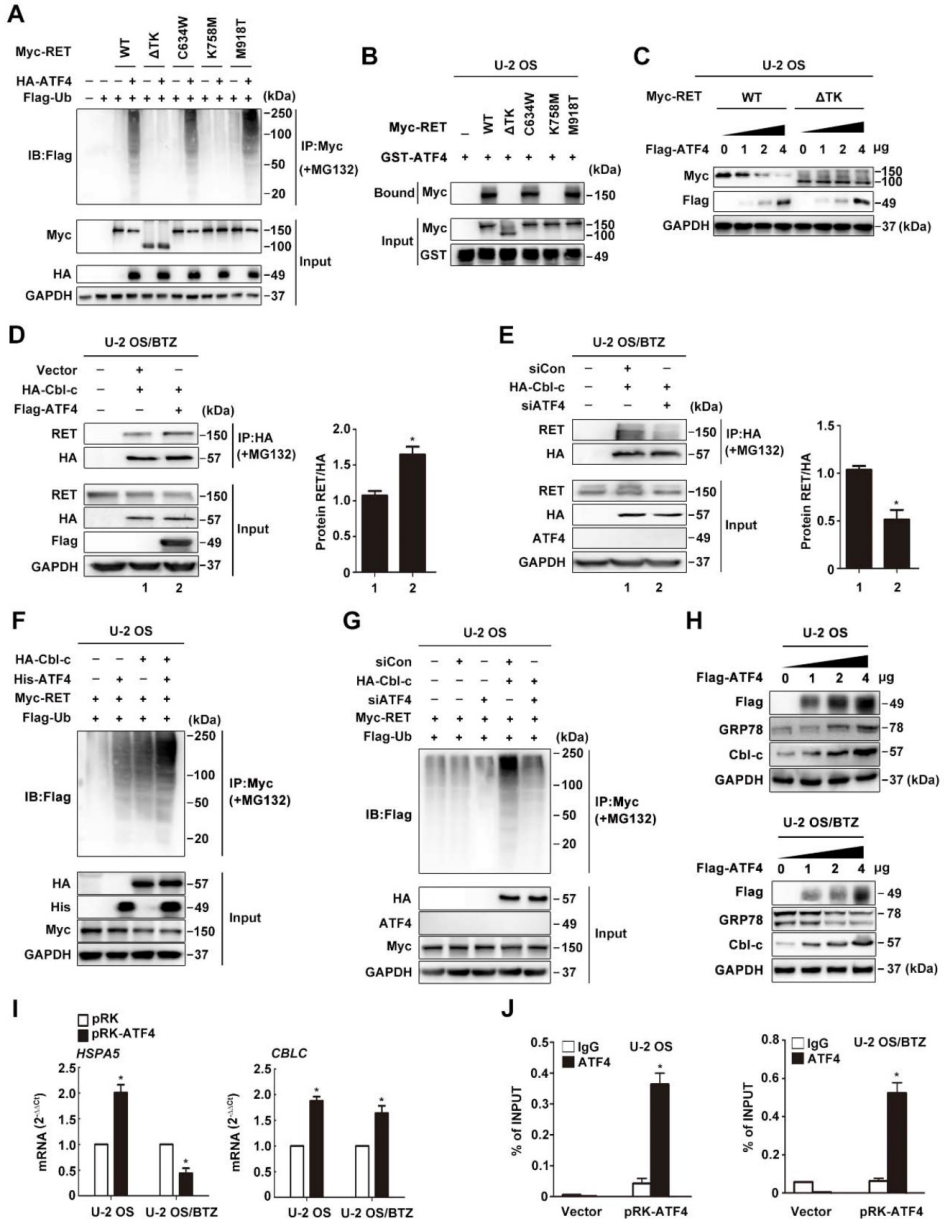
** The ATF4-Cbl-c-RET complex leads to RET degradation**. **A,** ATF4 promotes *in vivo* ubiquitination of RET but not ΔTK RET. Expression vectors encoding HA-ATF4, Myc-RET mutants and FLAG-ubiquitin were transfected into U-2 OS cells as indicated. The cell lysates were immunoprecipitated with anti-Myc antibody, and polyubiquitinated RET was detected by immunoblotting with anti-FLAG antibodies (upper panel). Expression level of each protein was assessed by anti-Myc, anti-HA, and anti-GAPDH antibodies (lower panel). **B,** Detection of the interaction between ATF4 and RET *in vitro*. Glutathione-agarose beads containing GST or GST-ATF4 were incubated with whole-cell extracts derived from U-2 OS cells expressing WT RET or ΔTK, C634W, K758M and M918T mutants. **C,** U-2 OS cells were transiently transfected with expression vectors encoding WT ATF4, WT RET, and ΔTK RET. After 24 h, the cell lysates were analysed by western blotting using antibodies against the indicated epitope tags. **D and E,** Coimmunoprecipitation of FLAG-ATF4 and HA-Cbl-c in U-2 OS/BTZ cells treated with 20 μM MG132 for 4 h. Overexpression of ATF4 increased the binding between RET and HA-Cbl-c (**D**), whereas knockdown of ATF4 decreased their binding (**E**). **F and G,** U-2 OS cells were cotransfected with HA-Cbl-c, Myc-RET, and FLAG-ubiquitin, and His-ATF4 (**F**) or siATF4 (**G**), and protein extracts were immunoprecipitated. Ubiquitination of RET was measured with an anti-Myc antibody. Cell lysates were immunoblotted with the indicated antibodies. **H,** U-2 OS and U-2 OS/BTZ cells transfected with increasing concentrations of the FLAG-ATF4 expression vector for 24 h were immunoblotted for endogenous proteins with the indicated antibodies.** I,** Expression of *HSPA5* and *CBLC* in control vector- and FLAG-ATF4-transfected OS and OS/BTZ cells analysed by qRT-PCR. **J,** ATF4 binds to the 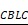 promoter. U-2 OS and U-2 OS/BTZ cells were transfected with ATF4 or control vector. Chromatin immunoprecipitation was performed using either a control IgG antibody or an antibody against ATF4. PCR primers were designed to amplify the specific *CBLC* promoter fragment spanning from -382 to +1. Primers for the *DDIT3* promoter were used as a positive control. Bars represent the mean ± SD, *P < 0.05.

Interestingly, TK-truncated RET (RET-ΔTK) and RET-K758M were not readily ubiquitinated; however, wild-type RET (WT), RET-C634W and RET-M918T were substantially ubiquitinated under the same conditions (**Figure 6A and S5B**). These results were confirmed by performing a GST pull-down assay in which WT RET, RET-C634W and RET-M918T were found to interact with ATF4, while RET-ΔTK and RET-K758M failed to interact with ATF4 (**Figure 6B and S5C**), suggesting that the ubiquitination sites on RET are located in the TK domain, and this interaction is dependent on the kinase activity but not constitutive autophosphorylation activity or conformational change in RET 44. We also found that the protein stability of WT RET rather than RET-ΔTK was decreased by ATF4 expression in a dose-dependent manner (**Figure 6C and S5D**), suggesting that the TK region of RET is associated with RET degradation.

### Cbl-c is recruited by ATF4 to ubiquitinate RET

Previously, Cbl-c, a RING finger ubiquitin ligase, was reported to be critical for the negative regulation of signalling by activated RET 43, 44. We observed a tight interaction between *CBLC* and *RET* via analysing the genes corresponding to RET degradation in the STRING database (**Figure 4I**), but the coexpression of the genes was not identified in *Homo sapiens* (**Figure S5E**). Therefore, we hypothesized that ATF4 might affect Cbl-c-dependent ubiquitination and degradation of RET. The interaction between RET and Cbl-c was analysed by immunoprecipitation in MG132-treated U-2 OS/BTZ sublines after HA-Cbl-c and FLAG-ATF4 overexpression. An increasing amount of RET was found to coimmunoprecipitate with HA-Cbl-c after FLAG-ATF4 expression compared with that before (**Figure 6D**), whereas ATF4 knockdown in the presence of MG132 almost abolished this pattern of interaction (**Figure 6E**), further supporting that the ectopic expression of ATF4 increases the interaction between RET and Cbl-c. Consistently, the introduction of ATF4 markedly facilitated Cbl-c-mediated ubiquitination of RET (**Figure 6F and S5F**). ATF4 silencing dramatically reduced RET ubiquitination (**Figure 6G**). Ectopic expression of HA-Cbl-c together with FLAG-ATF4 and Myc-RET in HEK293T cells further demonstrated a complex formation among RET, ATF4 and Cbl-c (**Figure S5G**). Also, we found Cbl-c promoted the interaction between ATF4 and RET, whereas Cbl-c knockdown significantly reduced ATF4 binding to RET in HEK293T cells (**Figure S5H**). Taken together, these data suggest that cooperative binding of ATF4 and Cbl-c with RET is necessary for RET ubiquitination. In comparison with the parental cell lines, BTZ-resistant sublines had lower Cbl-c protein levels, allowing for the depletion of ATF4 (**Figure S5I**). To obtain deeper insights into the mechanism underlying the regulation of such degradation, we investigated whether ATF4 participates in Cbl-c transactivation 40. First, Cbl-c protein levels were monitored in ATF4-overexpressing U-2 OS and U-2 OS/BTZ cells. We found that Cbl-c expression was significantly increased by ATF4 introduction in a concentration-dependent manner (**Figure 6H**). At the transcriptional level, *Cbl-c* mRNA was increased after ATF4 overexpression (**Figure 6I**) and decreased after ATF4 knockdown (**Figure S5J**). These results were then confirmed by chromatin immunoprecipitation analysis, showing that ATF4 could activate *Cbl-c* transcription (**Figure 6J**). The present findings suggested that ATF4 transactivates its target gene *Cbl-c* and recruits it to bind to RET for degradation.

### GRP78 blocks ATF4-mediated RET ubiquitination

**Figure 7:**
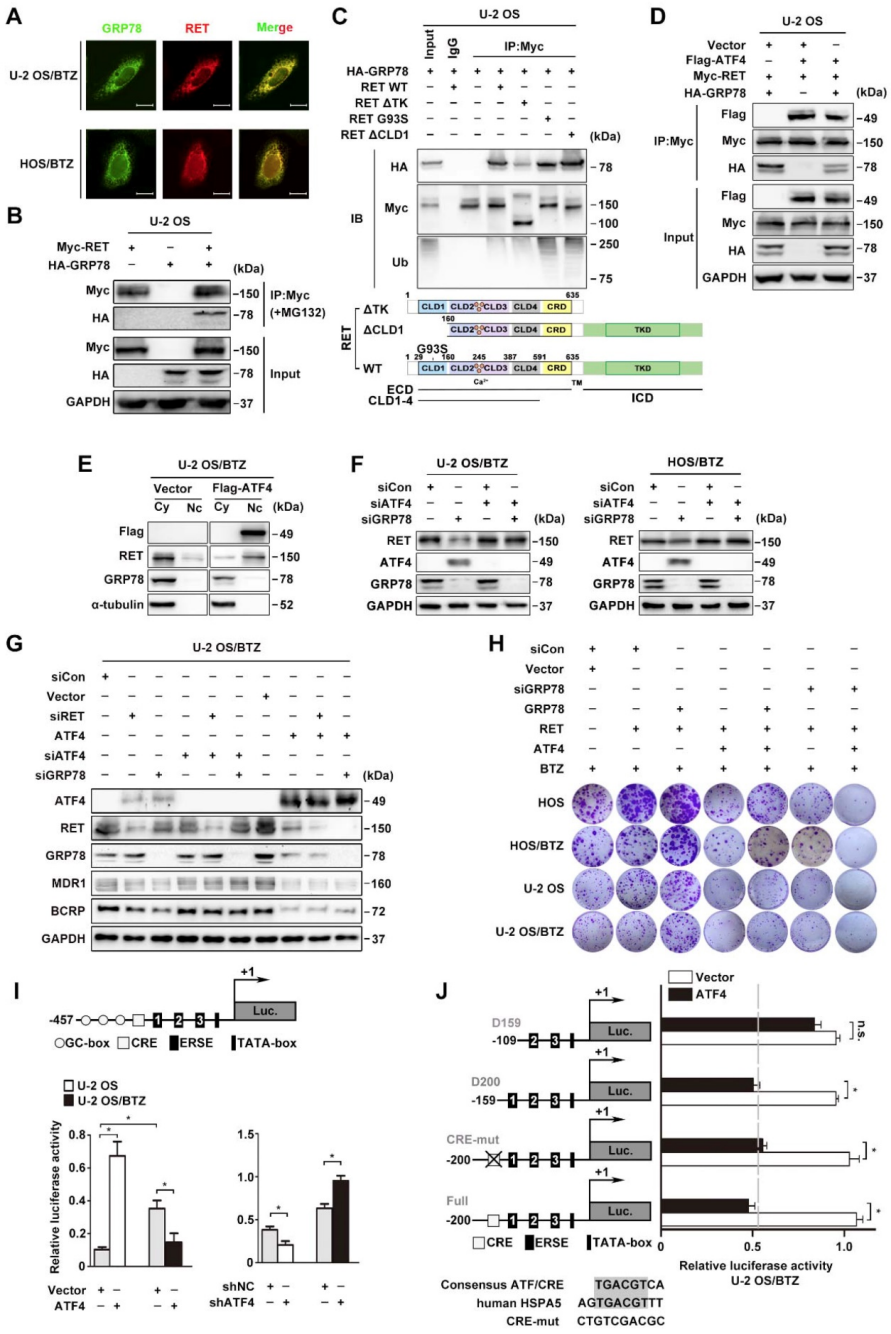
** GR*P*78 disrupts the binding of ATF4 to RET**. **A,** GRP78 colocalizes with RET in the cytoplasm. Confocal images of immunofluorescence staining of GRP78 and RET in human BTZ-resistant OS cells. Scale bars, 10 μm. **B,** U-2 OS cells were transiently transfected with Myc-RET or HA-GRP78 with or without the vector pcDNA3.1 for 2 days with MG132 (20 μM) treatment for 4 h, and whole-cell lysates were immunoprecipitated with anti-Myc antibody and blotted with the indicated antibodies. **C,** GRP78 interacts with the TK domain of RET to improve RET stability but interacts with the CLD1 domain for its degradation. Expression vectors encoding HA-GRP78 and Myc-RET mutants were transfected into U-2 OS cells as indicated. Cell lysates were immunoprecipitated with anti-Myc antibody, and the indicated proteins were detected by immunoblotting. Five percent of the HA-GRP78-transfected cell lysate for IP was used as input. **D,** U-2 OS cells were co-transfected with FLAG-ATF4, Myc-RET and HA-GRP78. Cell extracts were immunoprecipitated using an anti-Myc antibody and blotted with the indicated antibodies. **E,** Western blotting analysis of U-2 OS/BTZ cells showing RET downregulation and nuclear translocation after ATF4 overexpression in contrast to control vector. **F**, U-2 OS/BTZ and HOS/BTZ cells were transiently transfected with control (siCon), siATF4 or siGRP78 for 2 days. Protein lysates were harvested and subjected to immunoblotting analysis using the indicated antibodies. **G,** Equal amounts of protein lysates from a panel of U-2 OS/BTZ cells expressing control (siCon or vector), siRET, siATF4, siATF4 or ATF4 vectors were loaded for SDS-PAGE and western blotting analysis to detect the expression of the indicated proteins. GAPDH was used as a loading control. **H,** Colony formation assay was performed using paired OS and OS/BTZ cells transfected with control or siGRP78, GRP78, ATF4 and RET vectors with BTZ (100 nM) treatment for the first two days of the experiment. The cells were fixed, stained, and photographed after 14 days. **I,** Transcriptional activity of *HSPA5* in ATF4-expressing and control OS cell lines. Cells were transfected with the ATF4 expression plasmid and the *HSPA5* promoter (-457 to +1) luciferase reporter plasmid, and luciferase activity was determined 24 h after transfection. **J,** ATF4-mediated *HSPA5* promoter repression in BTZ-resistant OS requires the first box of ERSE. U-2 OS/BTZ cells were transfected with plasmids encoding the firefly luciferase gene driven by the illustrated promoter together with control vector (white bar) or plasmids encoding ATF4 (black bar). After 24 h, cells were harvested and analysed for luciferase activity. Data are shown as the mean ± SEM. Statistical significance was determined by Student's t-test. *P < 0.05; n.s., non-significant.

The stabilization of RET expression by the proteasome inhibitor MG132 occurred upon the simultaneous accumulation of GRP78 (**Figure 5A**). Confocal microscopy analysis revealed that GRP78 and RET have a strong subcellular colocalization primarily in the cytoplasm (**Figure 7A**). We also detected the expression of a specific GRP78-RET complex by coimmunoprecipitation in OS cells transfected with exogenous protein expression constructs (**Figure 7B and S6A**). GRP78 is known to be involved in the recognition of immature RET^ECD^ as a misfolded protein in secretory pathways 45. In agreement with this, both CLD1 deletion or expression of the immature form of G93S promoted GRP78-RET interaction and subsequent RET ubiquitination. However, GRP78 was also found to coimmunoprecipitate with the TK domain of RET, which appears to stabilize RET by yielding a less ubiquitinated RET complex (**Figure 7C**). Indeed, CHX chase assays showed that GRP78 overexpression in U-2 OS cells did not affect the mRNA level of *RET*, but prolonged the half-life of RET in U-2 OS/BTZ cells, compared with the control groups (**Figure S6B-C**). We further investigated whether this unique interaction allowed RET to escape from ATF4. The results showed that GRP78 significantly reduced the association of ATF4 with RET; ATF4 overexpression also reduced GRP78 binding to RET in U-2 OS and HEK293T cells (**Figure 7D and S6D**). Moreover, exogenous GRP78 prominently reversed the increase in the levels of ubiquitin conjugates induced by the ectopic expression of ATF4 in OS cells (**Figure S6E**). These results indicated a distinct role of GRP78 bound to RET in maintaining RET stability in contrast with the role of ATF4. In addition, we found that ATF4 overexpression in U-2 OS/BTZ cells induced the nuclear translocation and downregulation of RET, accompanied by the depletion of cytoplasmic GRP78 (**Figure 7E**).

We previously reported that GRP78 deficiency upregulated ATF4 expression in OS cells 7. To address whether the modulation of RET stability by ATF4 is dependent on GRP78 inhibition, we challenged OS/BTZ sublines with siRNAs against *ATF4* and *GRP78*. There was no significant decrease in RET protein upon the simultaneous suppression of ATF4 and GRP78, suggesting that ATF4 is a direct signal of RET degradation (**Figure 7F**). Consistent with these findings, we found that the loss of either RET or GRP78 downregulated the expression of MDR1 and BCRP associated with BTZ tolerance, as evidenced by ATF4 activation. A reversal of inhibition of chemoresistant proteins was also observed when ATF4 was silenced. In contrast, ATF4 overexpression decreased MDR1 and BCRP expression to basal levels (**Figure 7G**). These results supported the notion that high expression levels of ATF4 overcome BTZ tolerance in OS cells. A similar tendency was observed in colony formation assays where increases in *in vitro* proliferation were observed with concomitant induction of RET and GRP78 in OS and OS/BTZ cells (**Figure 7H**, lane 2 versus lane 3; **Figure S7A**). However, ATF4 overexpression was sufficient to alleviate the pro-proliferative activities of RET and GRP78, thus ameliorating resistance to BTZ, which was much more conspicuous when coupled with GRP78 knockdown (**Figure 7H**, lane 2 versus lane 4, lane 3 versus lane 5, and lane 4 versus lane 7, respectively; **Figure S7A**) highlighting the importance of ATF4 in accelerating OS progression during BTZ resistance.

### ATF4 exhibits distinct effects on the transcriptional regulation of GRP78

We observed concomitant upregulation of the GRP78 protein in U-2 OS cells displaying aberrant expression of ATF4 46. Intriguingly, we observed lower intracellular GRP78 levels in BTZ-resistant U-2 OS cells (**Figure 6H**). Similar changes were observed at the mRNA level (**Figure 6I**). Luciferase reporter assays and chromatin immunoprecipitation analyses further identified a contradictory transcriptional regulation of *HSPA5* by ATF4 in U-2 OS and U-2 OS/BTZ cells (**Figure 7I and S7B-C**). Furthermore, the DNA-binding domain (DBD) of ATF4 was required for both the activation and repression of *HSPA5* expression (**Figure S7D**) 47. ATF4 activates the *HSPA5* promotor by binding to an ATF/CRE sequence 48. To identify the *HSPA5* promoter elements responsible for ATF4-mediated transcriptional repression, we constructed a panel of luciferase plasmids containing promoter fragments with increasing 5'-deletions or ATF/CRE mutations and tested them after the transient cotransfection of ATF4 into U-2 OS/BTZ cells 49. There was no significant difference in *HSPA5* transcriptional activity between ATF4- and vector-expressing cells after the removal the first endoplasmic reticulum stress element (ERSE) (**Figure 7J**). However, ATF4 failed to transactivate* HSPA5* when the CRE element was mutated in U-2 OS cells (**Figure S7E**). These results suggested distinct mechanisms underlying the regulation of *HSPA5* transactivation by ATF4 in OS and OS/BTZ cells via independent binding domains.

### High-content screening for further clinical application

**Figure 8:**
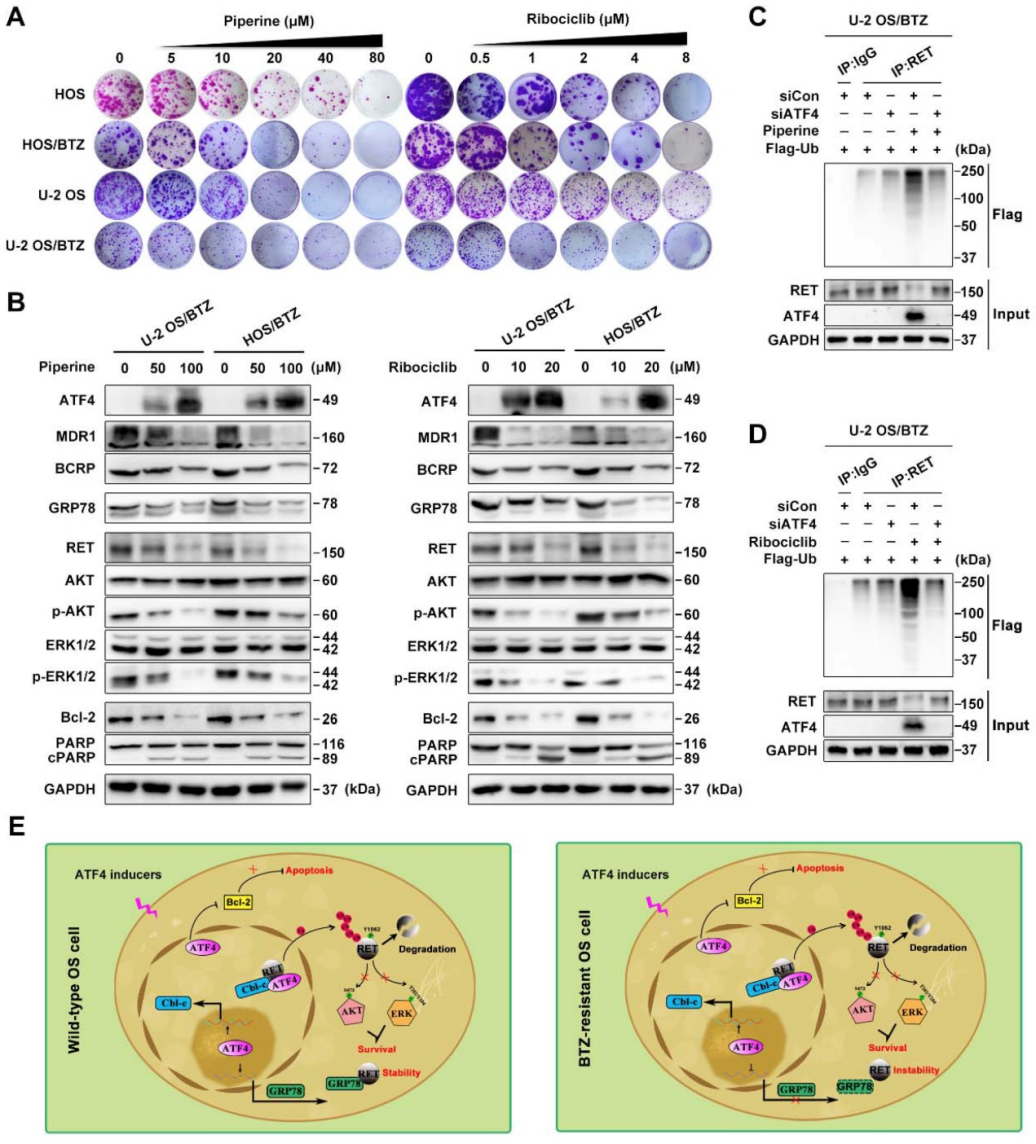
** ATF4 activator screening from a compound library**. **A,** Proliferation of control or piperine and ribociclib-treated OS and OS/BTZ cells was evaluated by colony formation assays. **B,** BTZ-resistant OS sublines were cultured in medium containing the indicated concentrations of piperine and ribociclib for 2 days, and lysates were blotted for the indicated proteins. **C and D,** ATF4 was knocked down in U-2 OS/BTZ cells, and then the cells were treated with piperine or ribociclib. Lysates were harvested from cells for immunoprecipitation with anti-RET antibody. IP samples were used to study ubiquitinated RET levels by western blotting with anti-FLAG antibody. **E,** Model indicating that the regulation of RET signalling by ATF4 leads to drug sensitivity in osteosarcoma. In wild-type OS cells, ATF4 promotes Cbl-c transcription and recruits it to synergistically to bind to RET by inducing the nuclear translocation of RET, thus resulting in the degradation of RET and inactivation of downstream signalling. Meanwhile, ATF4-transactivated GRP78 competitively interacts with RET, which is beneficial for RET stabilization. In contrast, ATF4 specifically transcriptionally inhibits GRP78 in BTZ-resistant OS cells, represents reinforced function of overwhelming RET signaling for the prevention of cancer malignancy drug resistance.

To expand the application of such mechanisms, we performed high-content screening to identify potential ATF4 activators in an FDA-approved drug library (1,452 compounds). We identified piperine and ribociclib as two potent hits with optimal effects on ATF4 induction in U-2 OS/BTZ cells (**Figure S7F**). Colony formation assays using OS and OS/BTZ cells treated with piperine and ribociclib indicated marked inhibition of *in vitro* proliferation (**Figure 8A and S8A**). Consistent with exogenous ATF4 overexpression, the compounds downregulated the expression of MDR1, BCRP and GRP78. The levels of RET and downstream effectors p-AKT and p-ERK1/2 were also repressed. Moreover, the Bcl-2 protein was inhibited, whereas PARP was activated (**Figure 8B**). Strikingly opposite results were obtained when ATF4 was knocked down (**Figure S7G**). We further demonstrated that ATF4 expression induced by piperine and ribociclib in OS/BTZ cells contributed to RET ubiquitination and degradation (**Figure 8C-D**). In addition, another 9 drugs were identified to be sufficient for upregulating ATF4, indicative of their potential in killing OS cells and overcoming chemoresistance (**Table S3**). Thus, these drugs might be used as promising agents for the treatment of OS patients.

## Discussion

The expression of ATF4 has been previously observed in osteosarcoma treated with BTZ, where it retains an apoptosis-inducing activity 7. Here, we demonstrated that the presence of upregulated ATF4 is beneficial for the growth delay of HOS xenografts and amplifies the apoptotic effects of BTZ, whereas ATF4 knockdown has opposite results. Dual AKT/ERK inhibition in response to ATF4 was observed in xenograft models. In normal bone tissues, ubiquitously expressed ATF4 of osteoblast cells (**Figure S1C**) is predominantly localized in the nucleus 50. In contrast, the present study revealed a loss of ATF4 in clinical osteosarcoma samples and established BTZ-resistant osteosarcoma sublines, which suggests that osteosarcoma cells attain drug resistance by orchestrating adaptive signalling to prevent ATF4 upregulation. Evidence indicates low *ATF4* expression in multiple cancers, such as human breast cancer and ovarian carcinoma (**Figure S1D-E**). Reduced expression of ATF4 is correlated with poor survival of patients with sarcoma. Therefore, the expression and nuclear localization of ATF4 is an important predictor of BTZ efficacy.

Long-term BTZ treatment often leads to acquired resistance in cancer. Our results showed a previously undescribed drug resistance mechanism in which GDNF-mediated RET phosphorylation at Y1062 promotes osteosarcoma progression by stimulating downstream AKT and ERK pathways. Unlike cisplatin chemoresistance in osteosarcoma, where GFRα1 regulates autophagy independent of the proto-oncogene *RET* kinase 44, RET was recognized as a functional driver of BTZ resistance in osteogenic sarcoma, suggesting RET as a target for the treatment of advanced osteosarcoma. Our study demonstrates that RET activation increases the expression of pro-survival factors and negatively affects BTZ-induced apoptotic cell death in OS and OS/BTZ cells. As BTZ is a well-characterized proteasome inhibitor 7, we hypothesized that the RET protein is preserved by long-term BTZ treatment in osteosarcoma via the development of a unique resistance mechanism. Indeed, RET has been shown to undergo rapid polyubiquitination and degradation upon activation by GDNF in neuronal homoeostasis 51. Emerging evidence suggests a bi-directional interplay between ATF4 and RET. RET prevents apoptosis through the inhibition of ATF4 activity during the pathogenesis of MTC 19. However, ATF4 has also been proposed to activate a negative-feedback loop, leading to the downregulation of RET expression while upregulating expression of pro-apoptotic genes in MTC 43. In this study, we show that pharmacological RET inhibition exhibited a synergistic effect with ATF4 in arresting the proliferation of OS and OS/BTZ cells, indicating that the induction of RET via ATF4 loss plays a critical role in the development of acquired BTZ resistance.

Previous studies suggest that Cbl-c can serve as a potential tumor suppressor 43. Notably, compared with that in parental cells, Cbl-c was downregulated in BTZ-resistant OS cells, accounting for the stability of RET. We showed that ATF4 increases Cbl-c expression at the transcriptional level in osteosarcoma. The ATF4-RET-Cbl-c interaction is required to prevent RET stabilization in OS/BTZ cells. GRP78 tends to interact with RET for degradation in the context of immature RET^ECD^ 45, indicating that the intact full-length RET protein exists in OS/BTZ sublines 19. Rather, ATF4 overexpression inhibited GRP78-RET binding, which induced the nuclear translocation of RET for degradation. These data indicate that the inverse correlation between the levels of ATF4 and GRP78 directly affects the stability and localization of RET in the nucleus. In addition, we identified distinct *HSPA5* transcriptional regulatory mechanisms by ATF4 in OS and OS/BTZ cells. Instead of transactivating *HSPA5* in OS cells 7, 8, ATF4 negatively regulates GRP78 transcription in BTZ-resistant OS cells, thus reinforcing the therapeutic effect of BTZ by the dual targeting of GRP78 and RET in the context of osteosarcoma chemoresistance (**Figure 8E**).

Taken together, our results reveal a novel molecular mechanism that underlies BTZ resistance to OS. The unfolded protein response regulator GRP78 and RTK member RET form a positive feedback loop to promote ATF4 loss and BTZ chemoresistance. Genetic or chemical inhibition of this feedback loop to enhance ATF4 expression may represent a novel approach to overcome chemoresistance. Thus, ATF4 may act as a promising biomarker to select patients for BTZ therapy or to identify therapeutic resistance, particularly in RET or GRP78-driven tumors.

## Supplementary Material

Supplementary figures and tables.

## Abbreviations

ABC: ATP-binding cassette
ATF4: activating transcription factor 4
BTZ: bortezomib
CBLC: Cbl proto-oncogene C
GDNF: glial cell line-derived neurotrophic factor
GRP78/HSPA5: heat shock protein family A member 5
OS: osteosarcoma
OS/BTZ: bortezomib-resistant human osteosarcoma
RET: ret proto-oncogene
RTK: receptor tyrosine kinase
