# Supplementary material for: ATF4 destabilizes RET through nonclassical GRP78 inhibition to enhance chemosensitivity to bortezomib in human osteosarcoma
Source: Theranostics. 2019 Aug 14;9(21):6334–53. doi: 10.7150/thno.36818 (PMC6735522; doi:10.7150/thno.36818)
Supplement: Supplementary file 1 — Supplementary figures and tables. [file thnov09p6334s1.pdf]

# ATF4 destabilizes RET through nonclassical GRP78 inhibition to enhance chemosensitivity to bortezomib in human osteosarcoma

Jie Luo[1,#], Yuanzheng Xia[1,#], Yong Yin[1], Jun Luo[1], Mingming Liu[1], Hao Zhang[1], Chao Zhang[1], Yucheng Zhao[1], Lei Yang[1], Lingyi Kong[1,*]

## Supplementary Figures

**Figure S1.**

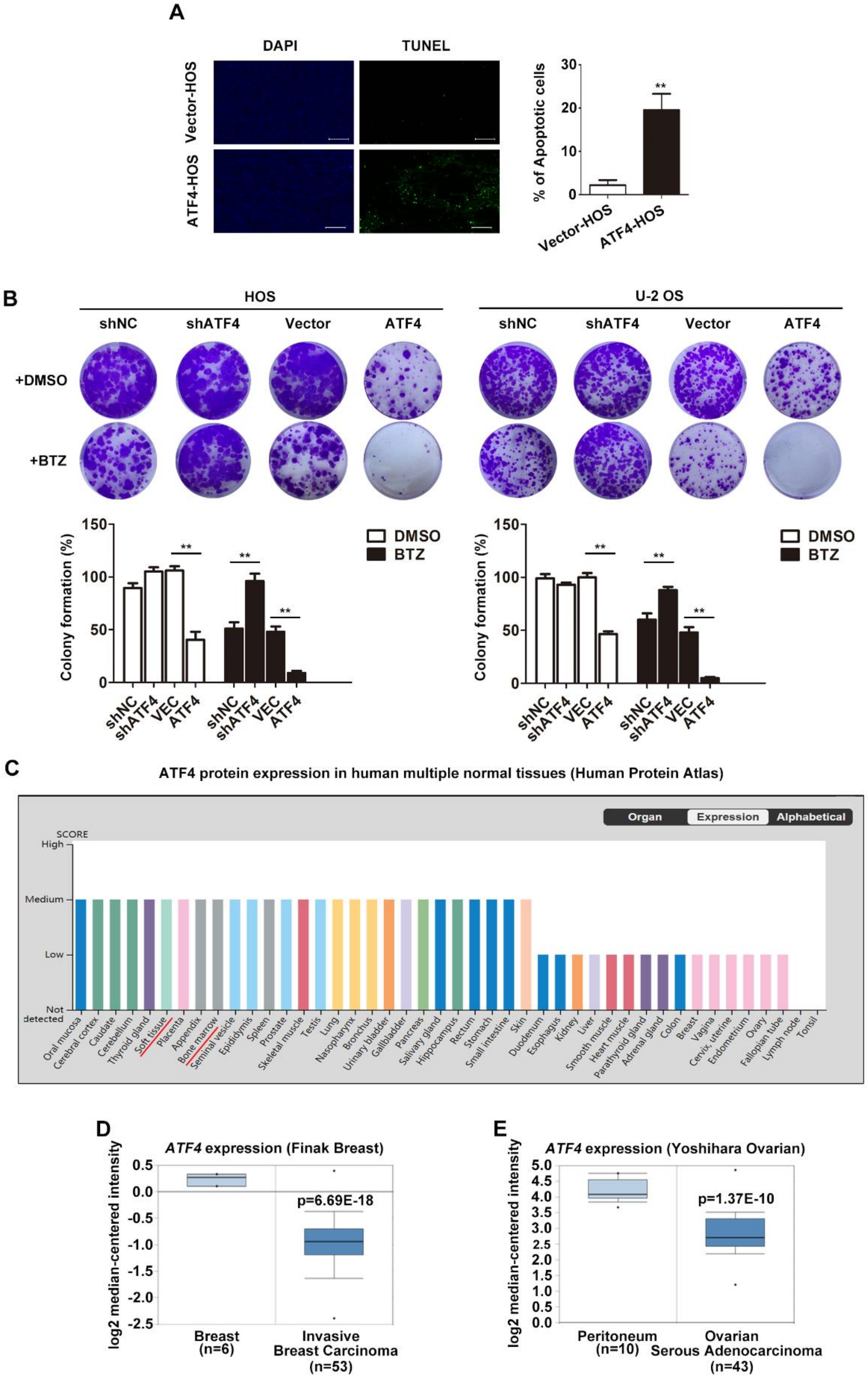
A
DAPI
TUNEL
Vector-HOS
ATF4-HOS
% of Apoptotic cells
**
Vector-HOS
ATF4-HOS
B
HOS
U-2 OS
shNC
shATF4
Vector
ATF4
+DMSO
+BTZ
Colony formation (%)
DMSO
BTZ
shNC
shATF4
VEC
ATF4
C
ATF4 protein expression in human multiple normal tissues (Human Protein Atlas)
Organ
Expression
Alphabetical
SCORE
High
Medium
Low
Not detected
Oral mucosa
Cerebral cortex
Caudate
Cerebellum
Thyroid gland
Soft tissue
Placenta
Appendix
Bone marrow
Seminal vesicle
Epididymis
Spleen
Prostate
Skeletal muscle
Testis
Lung
Nasopharynx
Bronchus
Urinary bladder
Gallbladder
Pancreas
Salivary gland
Hippocampus
Rectum
Stomach
Small intestine
Skin
Duodenum
Esophagus
Kidney
Liver
Smooth muscle
Heart muscle
Parathyroid gland
Adrenal gland
Colon
Breast
Vagina
Cervix, uterine
Endometrium
Ovary
Fallopian tube
Lymph node
Tonsil
D
ATF4 expression (Finak Breast)
log2 median-centered intensity
p=6.69E-18
Breast
(n=6)
Invasive
Breast Carcinoma
(n=53)
E
ATF4 expression (Yoshihara Ovarian)
log2 median-centered intensity
p=1.37E-10
Peritoneum
(n=10)
Ovarian
Serous Adenocarcinoma
(n=43)

**Figure S1. ATF4 is lowly expressed in multiple cancers and represents a prognostic marker.** **A,** Confocal images of TUNEL staining (green) in vector control and ATF4-expressing HOS xenograft tumor were shown. Nuclei were counterstained with Hoechst 33258 (blue). Scale bars represent 50 μm. **B,** Representative colony formation assay by monolayer culture with BTZ (100 nM) in ATF4-expressing or ATF4-deficient HOS and U-2 OS cells. Error bars represent mean ± SEM from three independent experiments (*$P < 0.05$, **$P < 0.01$). **C,** Bar graph illustrating *ATF4* expression levels by IHC in human normal tissues. Data were retrieved from Human Protein Atlas database (http://proteinatlas.org). **D and E,** Box plots showing differential *ATF4* mRNA expression levels between ovarian serous adenocarcinoma and normal peritoneum tissues, or invasive breast carcinoma and normal breast in Oncomine datasets. Error bars, SD.

## Figure S2.

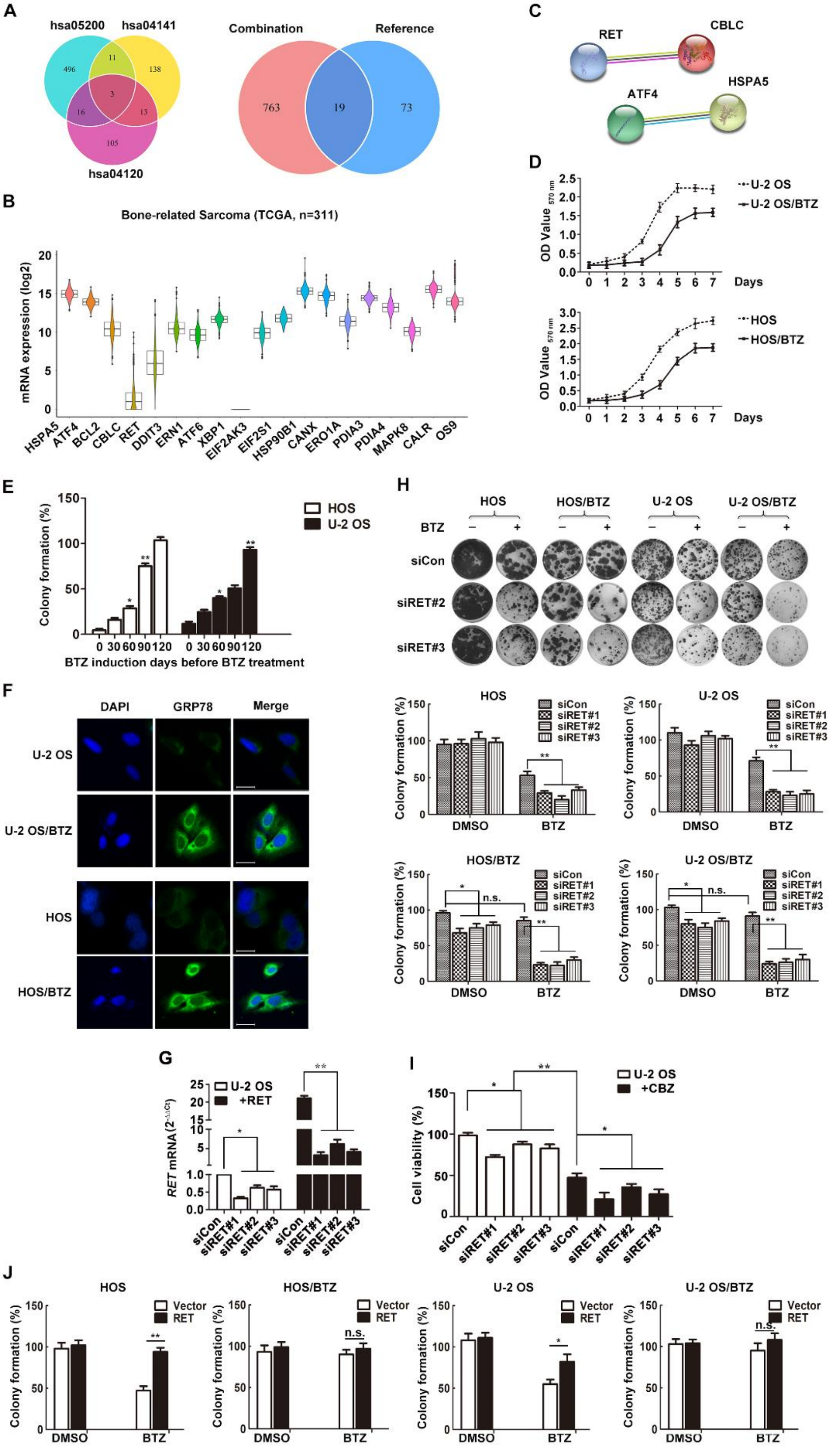

A
hsa05200
hsa04141
hsa04120
Combination
Reference
B
Bone-related Sarcoma (TCGA, n=311)
mRNA expression (log2)
C
RET
CBLC
ATF4
HSPA5
D
U-2 OS
U-2 OS/BTZ
HOS
HOS/BTZ
OD Value 570 nm
Days
E
HOS
U-2 OS
Colony formation (%)
BTZ induction days before BTZ treatment
F
DAPI
GRP78
Merge
U-2 OS
U-2 OS/BTZ
HOS
HOS/BTZ
G
U-2 OS
+RET
RET mRNA (2^-ΔΔCt)
siCon
siRET#1
siRET#2
siRET#3
H
HOS
HOS/BTZ
U-2 OS
U-2 OS/BTZ
BTZ
siCon
siRET#2
siRET#3
siRET#1
DMSO
n.s.
I
U-2 OS
+CBZ
Cell viability (%)
J
Vector
RET

**Figure S2. RET expression promotes osteosarcoma tumorigenesis and BTZ resistance. A,** Venn diagram indicating duplication among the three gene sets regarding cancer (hsa05200), protein processing in endoplasmic reticulum (hsa04141) and ubiquitin mediated proteolysis (hsa04120) in KEGG PATHWAY database. 782 nonredundant genes were obtained. A set of 19 genes were enriched for the three KEGG pathways mentioned above (Combination) and 92 genes (Reference) which were studied or interested by us and were confirmed the interaction in STRING database. **B,** Relative expression of the 19 genes were determined for all bone-related sarcoma samples (n = 311) in TCGA. **C,** The interactions of *HSPA5*, *ATF4*, *CBLC* and *RET* in STRING database. **D,** The BTZ-resistant subline U-2 OS/BTZ and HOS/BTZ was established. Cells were seeded into 96-well culture plates and incubated for 1-7 days, respectively. MTT assay was performed to measure the cell growth curve. **E,** Colony formation efficiencies were determined in BTZ-resistant models of U-2 OS and HOS cells treated with BTZ (100 nM). **F,** Immunofluorescence analysis of GRP78 protein (green) in OS/BTZ cells, in comparison to the parental cells. Nuclei were stained with Hoechst 33342 (blue). Images were taken using the high-content imaging system. Scale bars, 10 μm. **G,** Synthetic RET-siRNAs effectively suppressed endogenous *RET* mRNA in U-2 OS cells. Cells were transfected with 100 nM siRNA targeting RET (#1, 2, 3) or control siRNA (siCon), or cotransfected with ATF4 expression plasmid for 24 h. qRT-PCR quantitative analyses were performed to evaluate the efficiency of RET knockdown. **H,** RET colony formation-promoting activity. Control and RET knockdown (siRET#2 and #3) OS or OS/BTZ cells were cultured for 2 weeks with two-day BTZ (100 nM) treatment at the beginning. Then cells were fixed and stained. **I,** MTT assay of U-2 OS cells transfected with siRET (#1, 2, 3) or control siRNA in the presence of RTK inhibitor Cabozantinib (CBZ, 5 μM). **J,** Colony formation efficiencies were determined in RET-overexpressing OS or OS/BTZ cells with BTZ

(100 nM) treatment. Error bars represent mean ± SD from three independent experiments (**P* < 0.05, ***P* < 0.01； n.s., non-significant).

**Figure S3.**

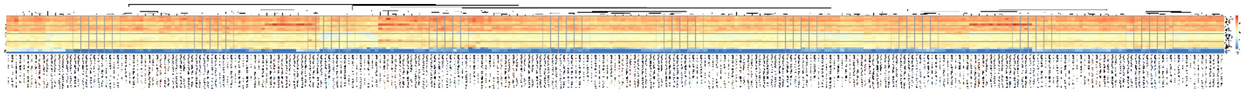

**Figure S3. Heatmap of 19 genes in normalized RNA-seq counts of 311 bone-related sarcoma samples in The Cancer Genome Atlas (TCGA) dataset.**

**Figure S4.**

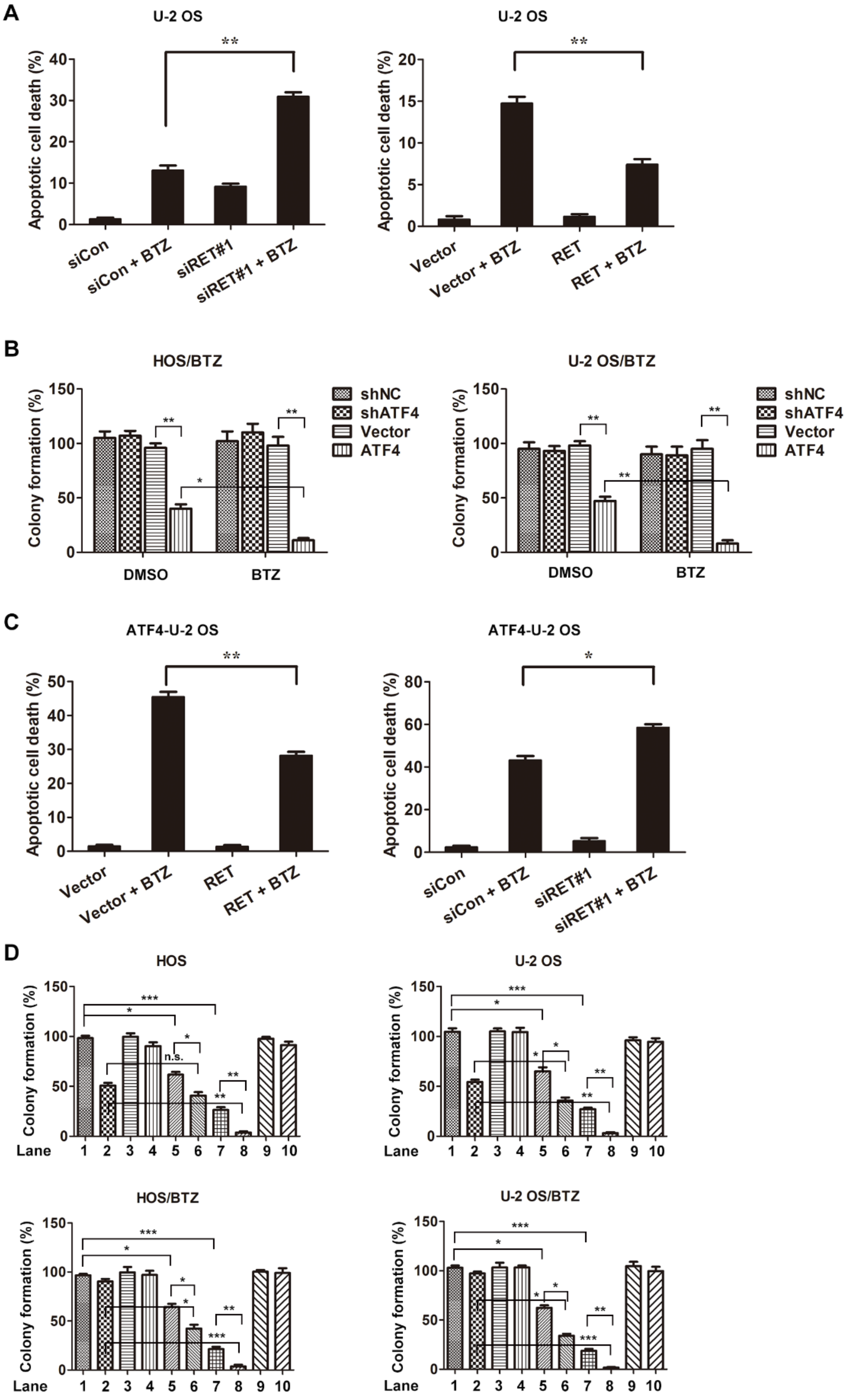
A
U-2 OS
Apoptotic cell death (%)
siCon
siCon + BTZ
siRET#1
siRET#1 + BTZ
Vector
Vector + BTZ
RET
RET + BTZ
B
HOS/BTZ
U-2 OS/BTZ
Colony formation (%)
shNC
shATF4
Vector
ATF4
DMSO
BTZ
C
ATF4-U-2 OS
D
HOS
U-2 OS
HOS/BTZ
U-2 OS/BTZ
Lane
n.s.

**Figure S4. The quantification for apoptotic and colony formation assays. A,** Quantification analysis of **Figure 3C**. U-2 $OS_{siRET\#1}$ and U-2 $OS_{siCon}$ cells or U-2 $OS_{RET}$ and U-2 $OS_{vector}$ cells were challenged by either vehicle or BTZ (100 nM), stained with fluorescein isothiocyanate (FITC)-conjugated Annexin V and propidium iodide (PI), and analysed by flow cytometry to evaluate the role of RET in the prevention of apoptosis. Significance is shown by **P < 0.01. **B,** Quantification analysis of **Figure 4A**. The functional phenotypes of retroviral shATF4 and ATF4-transfected OS/BTZ sublines are indicated by colony formation assays in 100 nM BTZ. The pGLV or EF1a vector was used as a control. The cells were fixed, stained, and photographed after 14 days. Error bars represent mean ± SD from three independent experiments (*$P$ < 0.05, **$P$ < 0.01). **C,** Quantification analysis of **Figure 4F**. Stable U-2 $OS_{ATF4}$ cells transfected with RET or siRET#1 were challenged with either vehicle or BTZ (100 nM), stained with FITC-conjugated Annexin V and PI, and analysed by flow cytometry to evaluate the role of RET in the prevention of apoptosis induction involving ATF4 overexpression. Significance is shown by *$P$ < 0.05 and **$P$ < 0.01. **D,** Quantification analysis of **Figure 4G**. Colony formation assays in OS and OS/BTZ cells, with either ATF4 or RET alterations mediated by the transient transfection of siRNAs or expression vectors, cultured in 100 nM for the first two days of the experiment. The cells were fixed, stained, and photographed after two weeks. Error bars represent mean ± SD from three independent experiments (*$P$ < 0.05, **$P$ < 0.01, ***$P$ < 0.001； n.s., non-significant).

Figure S5.

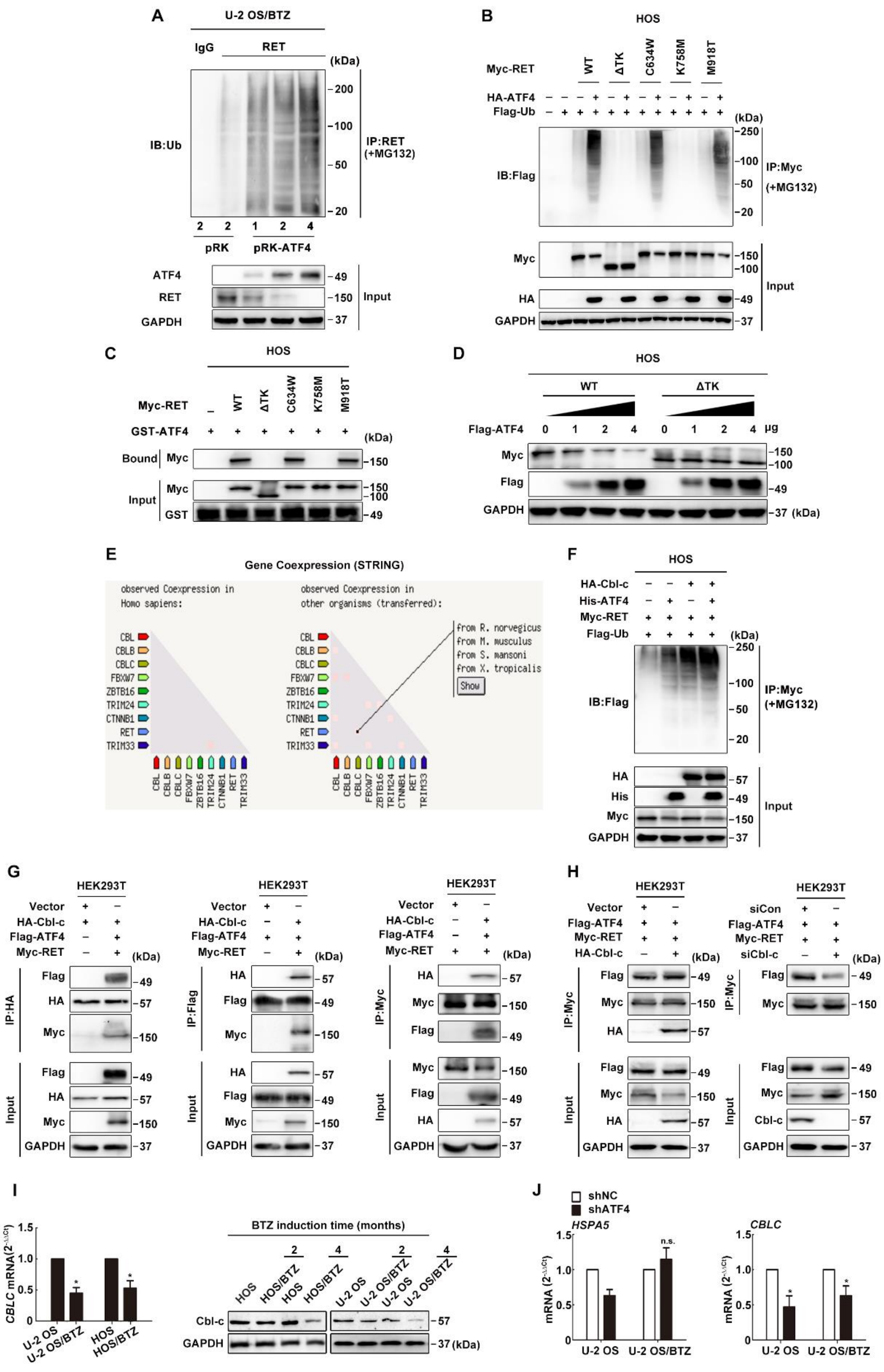

A
U-2 OS/BTZ
IgG
RET
(kDa)
IB:Ub
IP:RET (+MG132)
pRK
pRK-ATF4
ATF4
RET
GAPDH
Input
B
HOS
Myc-RET
WT
ΔTK
C634W
K758M
M918T
HA-ATF4
Flag-Ub
IB:Flag
IP:Myc (+MG132)
Myc
HA
GAPDH
Input
C
HOS
GST-ATF4
Bound
Input
GST
D
Flag-ATF4
μg
E
Gene Coexpression (STRING)
observed Coexpression in Homo sapiens:
observed Coexpression in other organisms (transferred):
from R. norvegicus
from M. musculus
from S. mansoni
from X. tropicalis
Show
CBL
CBLB
CBLC
FBXW7
ZBTB16
TRIM24
CTNNB1
RET
TRIM33
F
HA-Cbl-c
His-ATF4
Myc-RET
Flag-Ub
His
G
HEK293T
Vector
Flag-ATF4
IP:HA
IP:Flag
IP:Myc
H
siCon
siCbl-c
Cbl-c
I
CBLC mRNA(2^-ΔΔCt)
U-2 OS
U-2 OS/BTZ
HOS
HOS/BTZ
BTZ induction time (months)
J
shNC
shATF4
HSPA5
CBLC
mRNA (2^-ΔΔCt)
n.s.

**Figure S5. ATF4-Cbl-c-RET interaction accelerates RET turnover. A,** Ubiquitination profile of RET immunoprecipitated from U-2 OS/BTZ cells treated with increasing concentrations of ATF4 expression vector and incubated for 4 h in the presence of MG132 (20 μM). Immunoprecipitated samples were normalized for equivalent GAPDH loading. **B,** ATF4 promotes *in vivo* RET ubiquitination, but not ΔTK RET. Expressing vectors were transfected with HA-ATF4, Myc-RET mutants and FLAG-ubiquitin into HOS cells as indicated. The cell lysates were immunoprecipitated with anti-Myc antibody, and multiubiquitinated RET were detected by immunoblotting with anti-FLAG (upper panel). Expression level of each protein was assessed by anti-Myc, anti-HA, and anti-GAPDH (lower panel). **C,** Detection of the interaction between ATF4 and RET *in vitro*. Glutathione-agarose beads containing GST or GST-ATF4 were incubated with whole-cell extracts derived from HOS cells expressing WT RET, ΔTK, C634W, K758M and M918T mutants. **D,** HOS cells were transiently transfected with an expression vector for WT ATF4, WT RET, and ΔTK RET. After 24 h, the cell lysates were analyzed by western blot using antibodies against the indicated epitope tags. **E,** Coexpression profile of *RET* and its associated genes referred to ubiquitin process in STRING database. **F**, HOS cells were cotransfected with HA-Cbl-c, Myc-RET, and FLAG-ubiquitin, and His-ATF4, and protein extracts were immunoprecipitated. Ubiquitination of RET was measured with an anti-Myc antibody. Cell lysates were immunoblotted with the indicated antibodies. **G,** HEK293T cells were co-transfected with HA-Cbl-c, FLAG-ATF4, Myc-RET, or three vectors together. Cell extracts were IP using anti-HA, anti-FLAG or anti-Myc antibody and blotted with indicated antibodies. **H,** HEK293T cells were co-transfected with FLAG-ATF4 and Myc-RET, in the absence or presence of HA-Cbl-c or Cbl-c siRNAs. Cell extracts were pulled down using anti-Myc antibody and blotted with indicated antibodies. **I,** Expression of *CBLC* mRNA and protein in OS and OS/BTZ cells analyzed by qRT-

PCR and western blotting. **J,** Expression of *HSPA5* and *CBLC* in shNC control and ATF4-shRNA transfected OS and OS/BTZ cells analyzed by qRT-PCR. All values were mean ± SD relative to the basal condition, two-tailed unpaired Student's t-test, $*P < 0.05$, $**P < 0.01$; n.s., non-significant.

**Figure S6.**

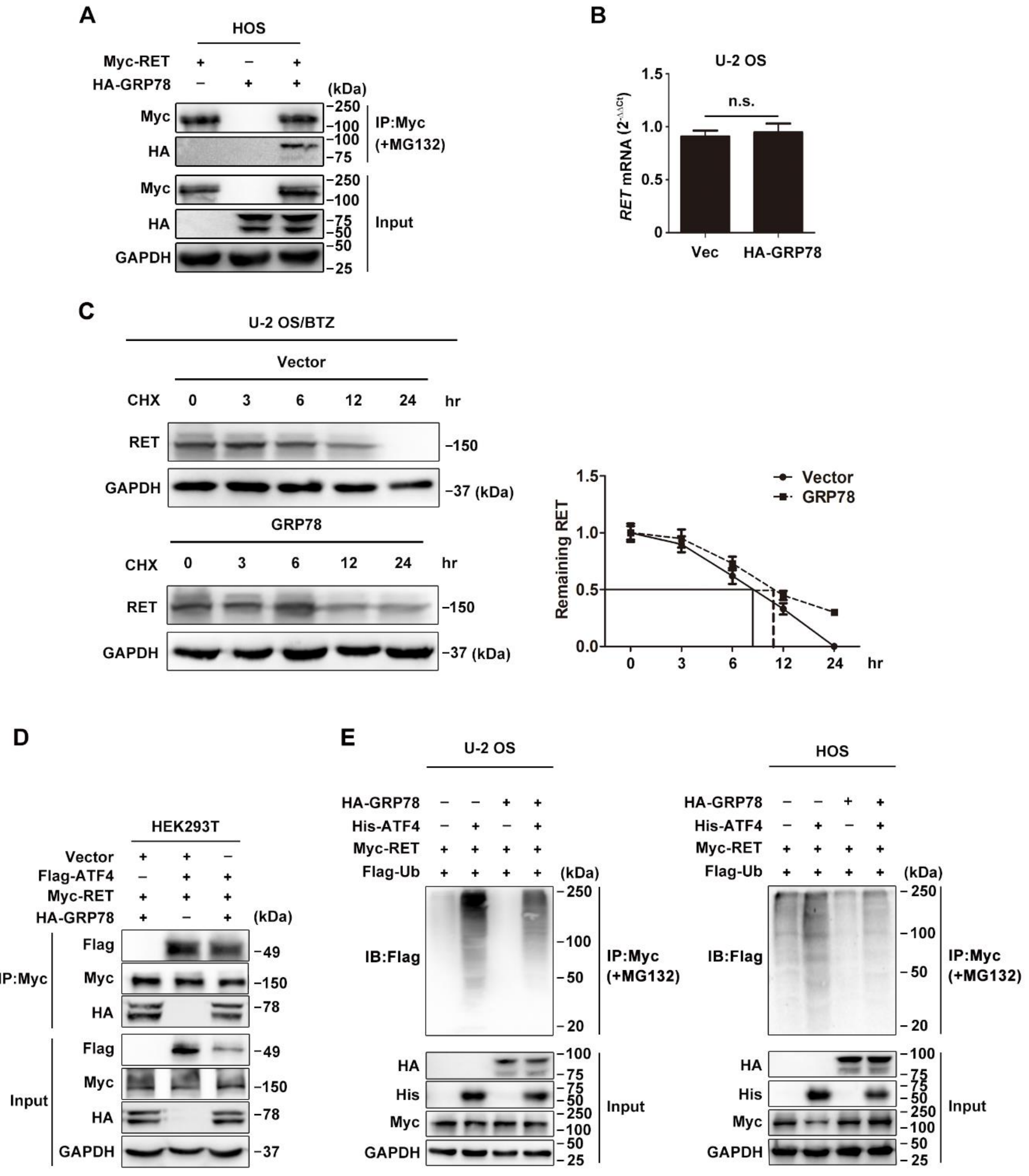


**Figure S6. GRP78 delays RET degradation through interfering with the binding of RET to ATF4**. **A,** HOS cells were transiently transfected with Myc-RET or HA-GRP78 with or without vector, pcDNA3.1(+) for 2 days with 4 h MG132 (20 μM) treatment and whole cell lysates were

immunoprecipitated with anti-Myc antibody and blotted with indicated antibodies. **B,** mRNA expression analysis of the *RET* gene in U-2 OS cells upon GRP78 expression using qRT-PCR. Data were expressed as the mean ± SD (n = 3) and analysed by two-tailed unpaired t-tests. n.s., non-significant. **C,** GRP78 prolongs the half-life of the RET protein. U-2/BTZ cells transfected with HA-GRP78 or control vector were treated with CHX (10 μM), and the expression of the indicated proteins was determined by immunoblotting at the indicated times. **D,** HEK293T cells were co-transfected with FLAG-ATF4 and Myc-RET or HA-GRP78 and Myc-RET. Cell extracts were immunoprecipitated using anti-Myc antibody and blotted with indicated antibodies. **E,** U-2 OS and HOS cells were cotransfected with Myc-RET, FLAG-ubiquitin, and His-ATF4 or HA-GRP78, and protein extracts were immunoprecipitated. Ubiquitination of RET was measured with an anti-Myc antibody. Cell lysates were immunoblotted with the indicated antibodies. Data are representative immunoblots of three independent assays.

**Figure S7.**

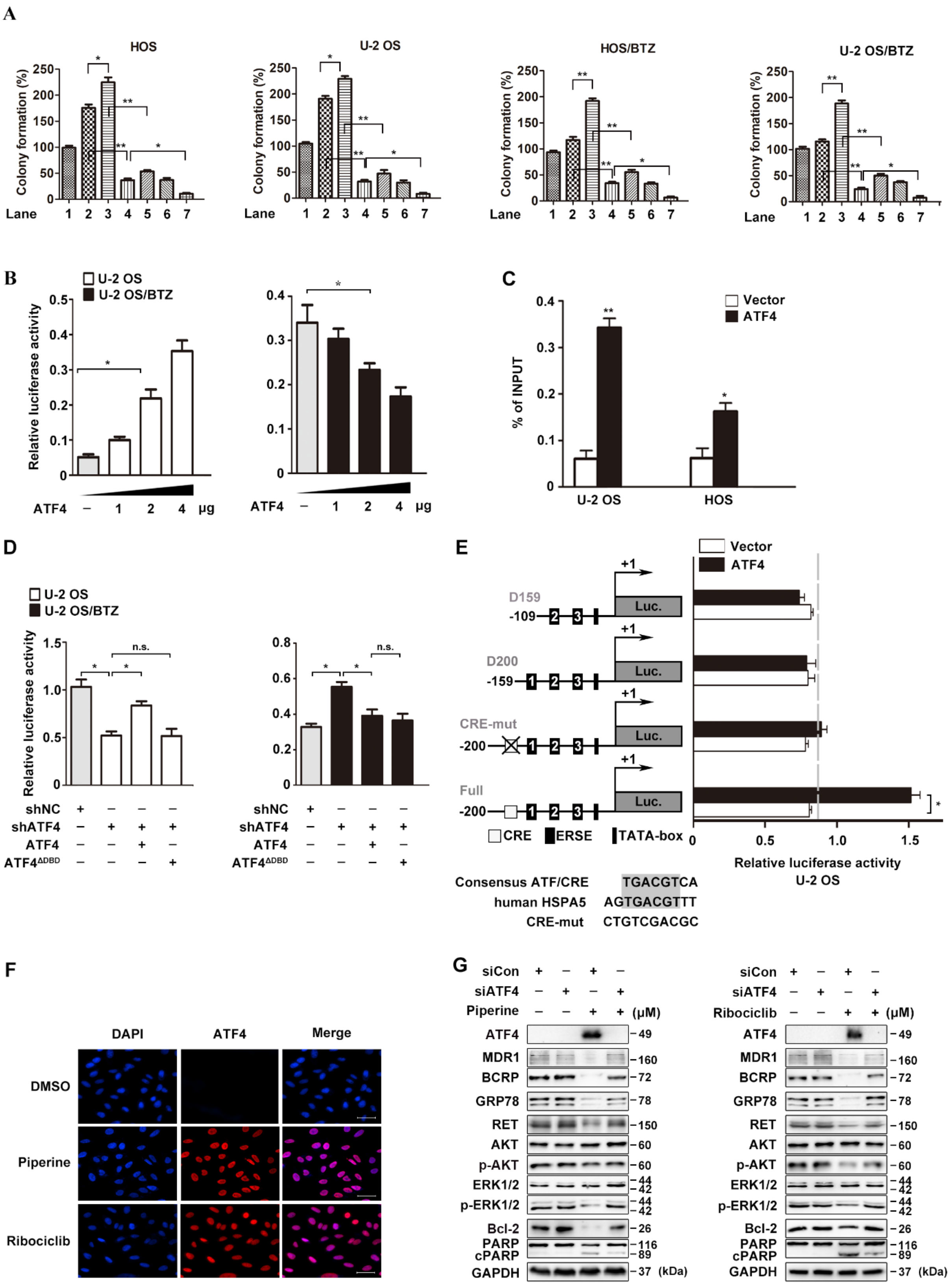

A
HOS
U-2 OS
HOS/BTZ
U-2 OS/BTZ
Colony formation (%)
Lane
1 2 3 4 5 6 7
B
U-2 OS
U-2 OS/BTZ
Relative luciferase activity
ATF4 – 1 2 4 μg
C
Vector
ATF4
% of INPUT
U-2 OS
HOS
D
U-2 OS
U-2 OS/BTZ
Relative luciferase activity
n.s.
shNC
shATF4
ATF4
ATF4ΔDBD
E
Vector
ATF4
+1
Luc.
D159
-109
D200
-159
CRE-mut
-200
Full
-200
CRE
ERSE
TATA-box
Relative luciferase activity
U-2 OS
Consensus ATF/CRE TGACGTCA
human HSPA5 AGTGACGTTT
CRE-mut CTGTCGACGC
F
DAPI
ATF4
Merge
DMSO
Piperine
Ribociclib
G
siCon
siATF4
Piperine
Ribociclib
(μM)
ATF4
MDR1
BCRP
GRP78
RET
AKT
p-AKT
ERK1/2
p-ERK1/2
Bcl-2
PARP
cPARP
GAPDH
(kDa)

**Figure S7. ATF4 exerts inverse transcriptional regulation of GRP78. A,** Quantification analysis of **Figure 7H**. Colony formation assay was performed using paired OS and OS/BTZ cells transfected with control or siGRP78, GRP78, ATF4 and RET vectors with BTZ (100 nM) treatment for the first two days of the experiment. The cells were fixed, stained, and photographed after 14 days. Error bars represent mean ± SD from three independent experiments (*$P$ < 0.05, **$P$ < 0.01). **B,** Relative luciferase activity driven by *HSPA5*promoter in U-2 OS and U-2 OS/BTZ cells. Cells were cotransfected with increasing concentrations of ATF4 plasmid and *HSPA5* promoter (-457 to +1)-luciferase reporter plasmid for 24 h, and then the luciferase values were determined. **C,** ATF4 binds to the *HSPA5* promoter. U-2 OS and HOS cells were transfected with ATF4 or vector control. Chromatin immunoprecipitation was performed using either a control IgG antibody or antibody against ATF4. PCR primers were designed to amplify the specific *HSPA5* promoter fragment spanning from -457 to +1. Primers for the *DDIT3* promoter were used as positive control. **D,** ATF4 transcriptionally regulates *HSPA5* through the DNA binding domain. Cells were transfected with the shRNA-ATF4, ATF4 or ΔDBD ATF4 expression plasmid and the *HSPA5* promoter (-457 to +1)-luciferase reporter plasmid, and luciferase activity was determined 24 h after transfection. **E,** ATF4-mediated *HSPA5* promoter repression in wild-type OS requires the CRE element. U-2 OS cells were transfected with the plasmid encoding the firefly luciferase gene driven by the illustrated promoter together with vector control (white bar) or the plasmid encoding ATF4 (black bar). After 24 h, cells were harvested and measured for luciferase activity. Data are shown as the mean ± SEM. Statistical significance was determined by Student's t-test. *$P$ < 0.05, **$P$ < 0.01; n.s., non-significant. **F,** Indirect immunofluorescence detecting the expression of ATF4 in U-2 OS/BTZ cells before subjected to Piperine or Ribociclib for 24 h. Images were taken using the high- content imaging system. Scale bars, 10 μm. **G,** OS/BTZ cells

transiently transfected with siCon or siATF4 were challenged with piperine or ribociclib for 24 h. Cell lysates were collected, and the indicated proteins were analysed. Data from representative immunoblots of three independent assays are shown.

**Figure S8.**

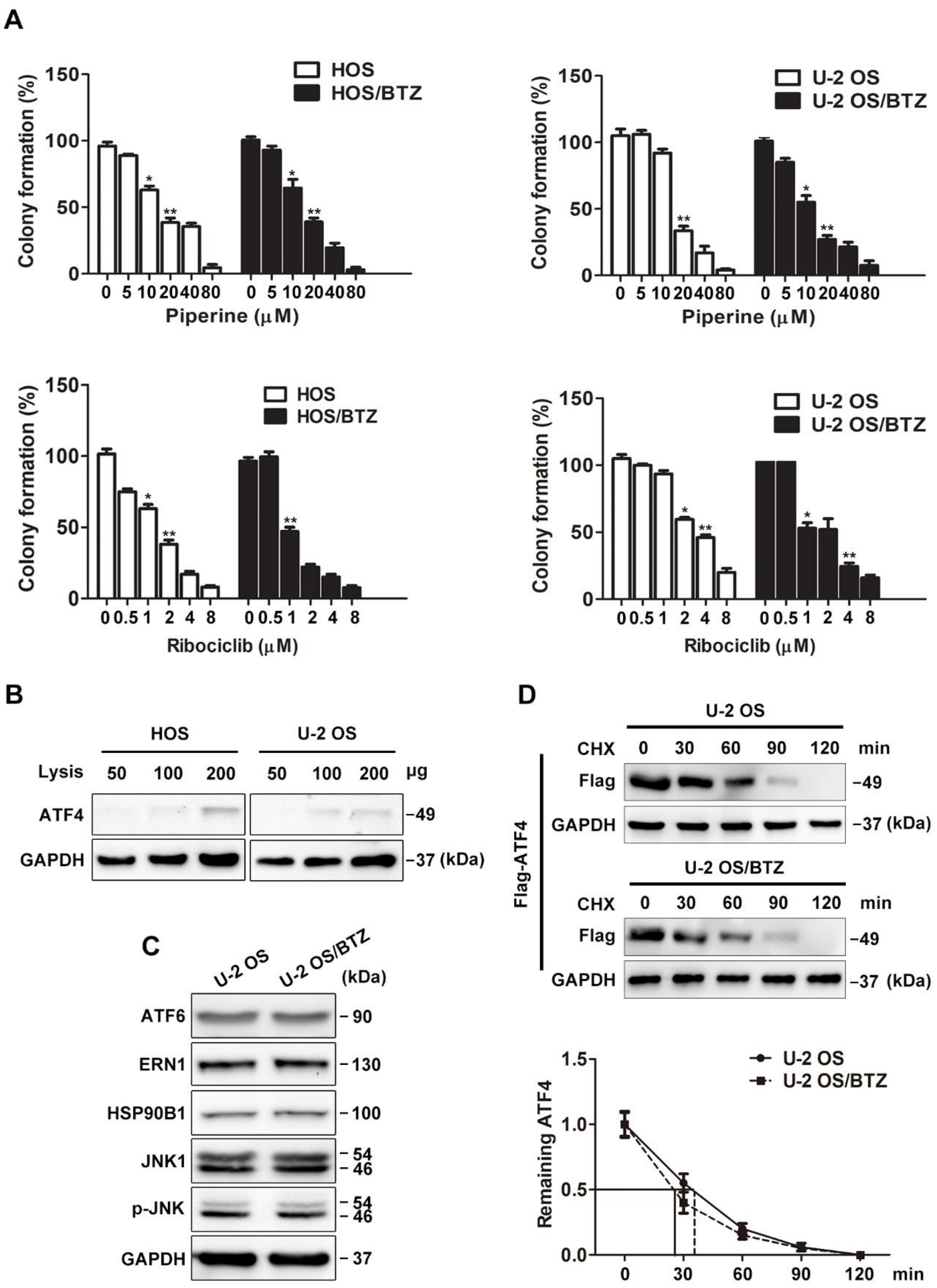
A
Colony formation (%)
HOS
HOS/BTZ
Piperine (μM)
U-2 OS
U-2 OS/BTZ
Ribociclib (μM)
B
HOS
U-2 OS
Lysis
50 100 200 50 100 200 μg
ATF4
GAPDH
-49
-37 (kDa)
C
U-2 OS
U-2 OS/BTZ
(kDa)
ATF6
ERN1
HSP90B1
JNK1
p-JNK
GAPDH
D
U-2 OS
U-2 OS/BTZ
CHX 0 30 60 90 120 min
Flag
GAPDH
Flag-ATF4
Remaining ATF4
min

**Figure S8. A,** Quantification analysis of **Figure 8A.** Proliferation of control or piperine and ribociclib-treated OS and OS/BTZ cells was evaluated by colony formation assays. Error bars represent mean ± SD from three independent experiments (**P* < 0.05, ***P* < 0.01). **B,** Western blotting analysis of the basal protein level of ATF4 with an increasing loading lysis from HOS and U-2 OS cells. **C,** Western blotting analysis of another 4 stress-related proteins ATF6, ERN1, HSP90B1and MAPK8 (JNK) in the parental and BTZ-resistant U-2 OS cells. **D,** The half-life of ATF4 protein in U-2 OS and U-2 OS/BTZ cell lines. Cells transfected with FLAG-ATF4 or control vector were treated with CHX (10 μM), and the expression of ATF4 was determined by immunoblotting at the indicated times. Data are representative immunoblots of three independent assays.

## 2. Supplementary Tables

**Table S1. Primary antibodies used in the study.**

| Antibody | Vendors | Catalogue No. | Applications | Dilution |
|---|---|---|---|---|
| ATF4 | CST | 11815 | WB | 1:1000 |
| GRP78 | CST | 3177 | WB | 1:1000 |
| RET | CST | 14556 | WB | 1:1000 |
| ABCB1 | CST | 12683 | WB | 1:1000 |
| MRP1 | CST | 14685 | WB | 1:1000 |
| Cleaved PARP (Asp214) | CST | 5625 | WB | 1:1000 |
| PARP | CST | 9532 | WB | 1:1000 |
| cCASP9 (Asp315) | CST | 20750 | WB | 1:1000 |
| cCASP3 (Asp175) | CST | 9664 | WB<br>IHC | 1:1000<br>1:1000 |
| p-AKT (Ser473) | CST | 4060 | WB | 1:2000 |
| AKT | CST | 4685 | WB | 1:1000 |
| p-ERK1/2 (Thr202/Tyr204) | CST | 8544 | WB | 1:1000 |
| ERK1/2 | CST | 12950 | WB | 1:1000 |
| Bcl-2 | CST | 4223 | WB | 1:1000 |
| Ubiquitin | CST | 3936 | WB | 1:1000 |
| GAPDH | CST | 5174 | WB | 1:5000 |
| α-tubulin | BOSTER | BM1452 | WB | 1:1000 |
| Cbl-c | Abcam | ab34750 | WB | 1:500 |
| BCRP | Abcam | ab24115 | WB | 1:1000 |
| RET | Abcam | ab134100 | IP<br>IF | 1:100<br>1:100 |
| ATF4 | Abcam | ab184909 | IF<br>IHC | 1:1000<br>1:100 |
| GRP78 | Abcam | ab108615 | IF | 1:200 |
| phospho-RET (Y1062) | Abcam | Ab51103 | WB | 1:1000 |
| RET (C-3) | Santa Cruz | sc-365943 | WB | 1:500 |
| GDNF(B-8) | Santa Cruz | sc-13147 | WB | 1:1000 |
| Bcl-2 | Bioss | bs-0032R | IHC | 1:500 |
| PCNA | Bioss | bs-0754R | IHC | 1:500 |
| JNK1 | CST | 3708 | WB | 1:1000 |
| p-JNK (Thr183/Tyr185) | CST | 9255 | WB | 1:1000 |
| GRP94 | CST | 20292 | WB | 1:1000 |
| ATF6 | CST | 65880 | WB | 1:1000 |
| IRE1α | CST | 3294 | WB | 1:1000 |
| FLAG-Tag | Sigma | F3165 | WB<br>IP | 1:2000<br>1:200 |
| FLAG-Tag | CST | 14793 | WB<br>IP | 1:2000<br>1:200 |
| Myc-Tag | Sigma | M4439 | WB<br>IP | 1:2000<br>1:200 |
| HA-Tag | Sigma | H6908 | WB<br>IP | 1:2000<br>1:200 |
| HA-Tag | CST | 2278 | WB | 1:2000 |

| | | | | |
|---|---|---|---|---|
| | | | **IP** | **1:200** |
| **His-Tag** | **CST** | **12698** | **WB** | **1:2000** |
| **GST-Tag** | **CST** | **2624** | **WB** | **1:2000** |

## Table S2. Sequences of real time PCR primers used in the study.

| **Gene** | **Forward primer (5'-3')** | **Reverse primer (5'-3')** |
|---|---|---|
| ***ATF4*** | ATGACCGAAATGAGCTTCCTG | GCTGGAGAACCCATGAGGT |
| ***RET*** | CAGAACCCTAGCCATAGCCG | AAAACGCACGAGGGAAAAGC |
| ***HSPA5*** | GAACGTCTGATTGGCGATGC | ACCACCTTGAACGGCAAGAA |
| ***CBLC*** | ACAGAAGGACGGCTTCTACC | AGTTCCCCTTTAGGAGTCTCA |
| ***BCL2*** | GAACTGGGGGAGGATTGTGG | CATCCCAGCCTCCGTTATCC |
| ***BCL-XL*** | CGGATTTGAATCTCTTTCTCTCCC | CGACCCCAGTTTACCCCATC |
| ***MCL-1*** | CTTTTGGCTACGGAGAAGGAG | GTCACAATCCTGCCCCAGTT |
| ***BAX*** | CCAGAGGCGGGGTTTCAT | CATCCTCTGCAGCTCCATGT |
| ***BAK*** | ACAGCCTGTTTGAGAGTGGC | GCAGGGAGGACATTGCAGTC |
| ***BAD*** | AGAGTTTGAGCCGAGTGAGC | ATCCCACCAGGACTGGAAGA |
| ***BID*** | CATGGACCGTAGCATCCCTC | AGCACCAGCATGGTCTTCTC |
| ***BIM*** | GTATTCGGTTCGCTGCGTTC | ACCTCCGTGATTGCCTTCAG |
| ***NOXA*** | TGCAGGACTGTTCGTGTTCA | TGAACTGTTTCTCCCCAGCC |
| ***PUMA*** | GAAAGGCTGTTGTGCTGGTG | AGGCTAGTGGTCACGTTTGG |
| **GAPDH** | GAAAGCCTGCCGGTGACTAA | AGGAAAAGCATCACCCGGAG |
| ***HSPA5* promoter (-457 to +1)** | GCGGATGGGGCGGATGTTAT | TCTTGCCAGCCAGTTGGGCA |
| ***HSPA5* promoter (Full, CRE-mut, D200, D159)** | TGGCGCCTTGTGACCCCGGG | TCTTGCCAGCCAGTTGGGCA |
| ***CBLC* promoter (-382 to +1)** | CCGCCTGCCTCGGCCTCCTA | TGGGAGCCTCGCGTGTGCCG |
| ***DDIT3* promoter (-400 to +1)** | AAGTTGCCTCTCCCCCTTCC | TCTCTGCAGTTGATCAGTC |

## Table S3. High-throughput screening of FDA-approved Drugs

| *Summary of the High Throughput Screen* | ATF4 Induction | Target(s) | Disease(s) |
|---|---|---|---|
| Piperine | ✓ | MDR1 | Cancer |
| Ribociclib | ✓ | CDK4/CDK6 | Cancer |
| Tozasertib | ✓ | Aurora A, B, C | Cancer |
| Decitabine | ✓ | DNMT | Cancer |
| Iloperidone | ✓ | D2/5-HT2 receptor | Schizophrenia |
| Bafetinib | ✓ | Bcr-Abl/Lyn | Cancer |
| Moxonidine | ✓ | I1-R | Hypertension |
| (+, -)-Octopamine HCl | ✓ | Dopamine receptor | Neuromodulation |
| S-Ruxolitinib | ✓ | JAK1/2 | Cancer |
| Alisertib | ✓ | Aurora A | Cancer |
| Dabrafenib | ✓ | BRAF | Cancer |

Summary of a high-throughput screening of 1,452 biologically active compounds approved by the FDA for ATF4 induction in BTZ-resistant U-2 OS cells. Immunofluorescence data are represented as the mean of triplicate samples ± SD and are representative of three independent experiments.

**Table S4. Software and Algorithms for statistics**

| | | |
|---|---|---|
| ***GraphPad Prism*** | GraphPad | https://www.graphpad.com/scientificsoftware/prism/ |
| ***Heatmap Illustrator*** | Wankun Deng, Yongbo Wang, Zexian Liu, Han Cheng and Yu Xue. PLoS One 2014 Nov 5;9(11):e111988 | http://hemi.biocuckoo.org/ |
| ***R Programming Language*** | R Project for Statistical Computing | https://www.r-project.org/ |
| ***R Studio*** | R-Studio | https://www.rstudio.com/ |
| ***data.table*** | CRAN - Package | https://CRAN.R-project.org/package=data.table |
| ***dplyr*** | CRAN - Package | https://CRAN.R-project.org/package=dplyr |
| ***pheatmap*** | CRAN - Package | https://cran.r-project.org/package=pheatmap |
| ***gridGraphics*** | CRAN - Package | https://CRAN.R-project.org/package=gridGraphics |
| ***futile.logger*** | CRAN - Package | https://CRAN.R-project.org/package=futile.logger |
| ***VennDiagram*** | CRAN - Package | https://CRAN.R-project.org/package=VennDiagram |
| ***ggplot2*** | CRAN - Package | https://CRAN.R-project.org/package=ggplot2 |
| ***R.devices*** | CRAN - Package | https://CRAN.R-project.org/package=R.devices |
| ***corrplot*** | CRAN - Package | https://CRAN.R-project.org/package=corrplot |
| ***BiocManager*** | CRAN - Package | https://CRAN.R-project.org/package=BiocManager |
| ***AnnotationDbi*** | Pagès H, Carlson M, Falcon S, Li N (2018). AnnotationDbi: Annotation Database Interface. R package version 1.44.0. | http://bioconductor.org/packages/AnnotationDbi/ |
| ***org.Hs.eg.db*** | Carlson M (2018). org.Hs.eg.db: Genome wide annotation for Human. R package version 3.7.0. | http://bioconductor.org/packages/org.Hs.eg.db/ |
| ***Microsoft Office*** | Microsoft Corporation | https://www.office.com/ |
